# Slitrk4 is required for the development of inhibitory neurons in the fear memory circuit of the lateral amygdala

**DOI:** 10.3389/fnmol.2024.1386924

**Published:** 2024-04-26

**Authors:** Yoshifumi Matsumoto, Hideki Miwa, Kei-ichi Katayama, Arata Watanabe, Kazuyuki Yamada, Takashi Ito, Shinsuke Nakagawa, Jun Aruga

**Affiliations:** ^1^Laboratory for Behavioral and Developmental Disorders, RIKEN Brain Science Institute, Wako-shi, Japan; ^2^Department of Genetic and Behavioral Neuroscience, Gunma University Graduate School of Medicine, Maebashi, Japan; ^3^Department of Neuropsychopharmacology, National Institute of Mental Health, National Center of Neurology and Psychiatry, Tokyo, Japan; ^4^Department of Medical Pharmacology, Nagasaki University Institute of Biomedical Sciences, Nagasaki, Japan; ^5^Support Unit for Animal Experiments, RIKEN Brain Science Institute, Wako-shi, Japan; ^6^Department of Biochemistry, Nagasaki University Institute of Biomedical Sciences, Nagasaki, Japan

**Keywords:** Slitrk4, amygdala, fear memory, neural plasticity, inhibitory neuron, cell type specification, gene targeting, posttraumatic stress disorder

## Abstract

The Slitrk family consists of six synaptic adhesion molecules, some of which are associated with neuropsychiatric disorders. In this study, we aimed to investigate the physiological role of Slitrk4 by analyzing Slitrk4 knockout (KO) mice. The Slitrk4 protein was widely detected in the brain and was abundant in the olfactory bulb and amygdala. In a systematic behavioral analysis, male Slitrk4 KO mice exhibited an enhanced fear memory acquisition in a cued test for classical fear conditioning, and social behavior deficits in reciprocal social interaction tests. In an electrophysiological analysis using amygdala slices, Slitrk4 KO mice showed enhanced long-term potentiation in the thalamo-amygdala afferents and reduced feedback inhibition. In the molecular marker analysis of Slitrk4 KO brains, the number of calretinin (CR)-positive interneurons was decreased in the anterior part of the lateral amygdala nuclei at the adult stage. In *in vitro* experiments for neuronal differentiation, Slitrk4-deficient embryonic stem cells were defective in inducing GABAergic interneurons with an altered response to sonic hedgehog signaling activation that was involved in the generation of GABAergic interneuron subsets. These results indicate that Slitrk4 function is related to the development of inhibitory neurons in the fear memory circuit and would contribute to a better understanding of osttraumatic stress disorder, in which an altered expression of Slitrk4 has been reported.

## 1 Introduction

Fear learning is an essential brain function for mammals to avoid predictable risky situations where insulting stimuli are correlated with the context and cues of the external environment. However, impaired control of fear memory in humans, particularly excessive fear against various subjects, can result in pathophysiological conditions such as agoraphobia, social phobia, other specific phobias, and posttraumatic stress disorder (PTSD). Clarification of the mechanism underlying fear learning control in mammalian model animals is one of the major challenges in current neurobiology.

Associative fear learning in rodents has been well-studied in terms of context-dependent and cue-dependent conditioned stimuli. In a typical experimental protocol, the animals are first transferred to a chamber with discernible features and then given an electric foot shock (unconditioned noxious stimulus) preceded by short sound stimuli (cued conditional stimuli). After conditioning by the coupled conditioned and unconditioned stimuli, animals were exposed to the chamber (context) or the sound (cue) and tested for the posture of alert (freezing response) quantitatively.

Recent studies have revealed neural circuits and their critical molecular components for fear learning. The amygdala is an important brain region for fear learning (LeDoux, 2000). In the case of the rodent fear conditioning experiment, the acquisition of cue-dependent fear memory requires the amygdala neural circuit (Ehrlich et al., 2009; Johansen et al., 2011; Duvarci and Pare, 2014). Both auditory input and noxious input target pyramidal neurons in the lateral nuclei of the amygdala (LA). Long-term potentiation occurs in the medial geniculate body (MGB)-LA pyramidal neuron synapses by the simultaneous stimulation of auditory and noxious inputs, providing a cellular basis for fear memory acquisition. It is known that LA local inhibitory interneurons innervate the pyramidal cells and control the MGB-LA synapses through feedback and feedforward inhibition (Samson and Pare, 2006; Ehrlich et al., 2009). However, the molecular components that establish LA local inhibitory neuronal circuits are not fully understood.

Here we show that Slitrk4, a member of the Slitrk family protein, is an essential gene for the proper development of the amygdala inhibitory neural circuit. Slitrk proteins are required for synaptogenesis in the murine hippocampus and retina (Takahashi et al., 2012; Tekin et al., 2013), innervation and survival of inner ear sensory neurons (Katayama et al., 2009), and proper behaviors (Katayama et al., 2010; Matsumoto et al., 2011). Human SLITRK family genes are associated with neurological disorders, such as Tourette's syndrome and obsessive-compulsive disorder (SLITRK1) and sensory deafness/myopia comorbidity (SLITRK6) (Abelson et al., 2005; Tekin et al., 2013). The SLITRK family consists of six dual leucine-rich repeat transmembrane proteins (Slitrk1 to Slitrk6) in humans and mice, sharing neurite development-controlling and synaptogenic activities (Aruga and Mikoshiba, 2003; Aruga et al., 2003; Takahashi et al., 2012). Both human SLITRK4 and mouse Slitrk4 are located on X chromosome (Aruga and Mikoshiba, 2003; Aruga et al., 2003). SLITRK4 can rescue neurite outgrowth deficits in myotonic dystrophy type 1 (DM1) patient-derived cells, and its expression is downregulated in the neural cells and brains of DM1 patients (Marteyn et al., 2011), suggesting its clinical importance in DM1. However, no studies have addressed the functional significance of Slitrk4 with genetic evidence.

We investigated the physiological role of Slitrk4 by analyzing Slitrk4 knockout (KO) mice. We firstly surveyed brain function-associated abnormalities by an unbiased behavioral test battery. Because the mice displayed enhanced cue-based fear conditioning, possible basis underlying the enhanced fear memory was explored by electrophysiology and molecular marker analyses. The results suggested that an impaired feedback regulation of the LA neural circuit and deficits of interneuron subpopulation development occur in Slitrk4 KO brains.

## 2 Materials and methods

### 2.1 Animals

All animal experiments were approved by the Animal Experiment Committees at the RIKEN Brain Science Institute and Animal Care and Use Committee of Nagasaki University (1803271441) and carried out following the guidelines for animal experimentation at RIKEN and Nagasaki University.

Adult male mice were used for all analyses to avoid effects of estrous cycles on behavioral phenotypes in females (Meziane et al., 2007). For behavioral experiments, mice were housed individually before being transferred to the behavioral laboratory. They were kept in the laboratory during the behavioral analysis. The light conditions were 12:12 h. The laboratory was air-conditioned and maintained temperature and humidity within ~22–23°C and 50–55%, respectively. Food and water were freely available, except during the experiment. All behavioral experiments were conducted in the light phase (9:00–18:00), and the starting time of the experiments was kept constant. All experiments were carried out using experimentally naïve mice.

**Figure 1:**
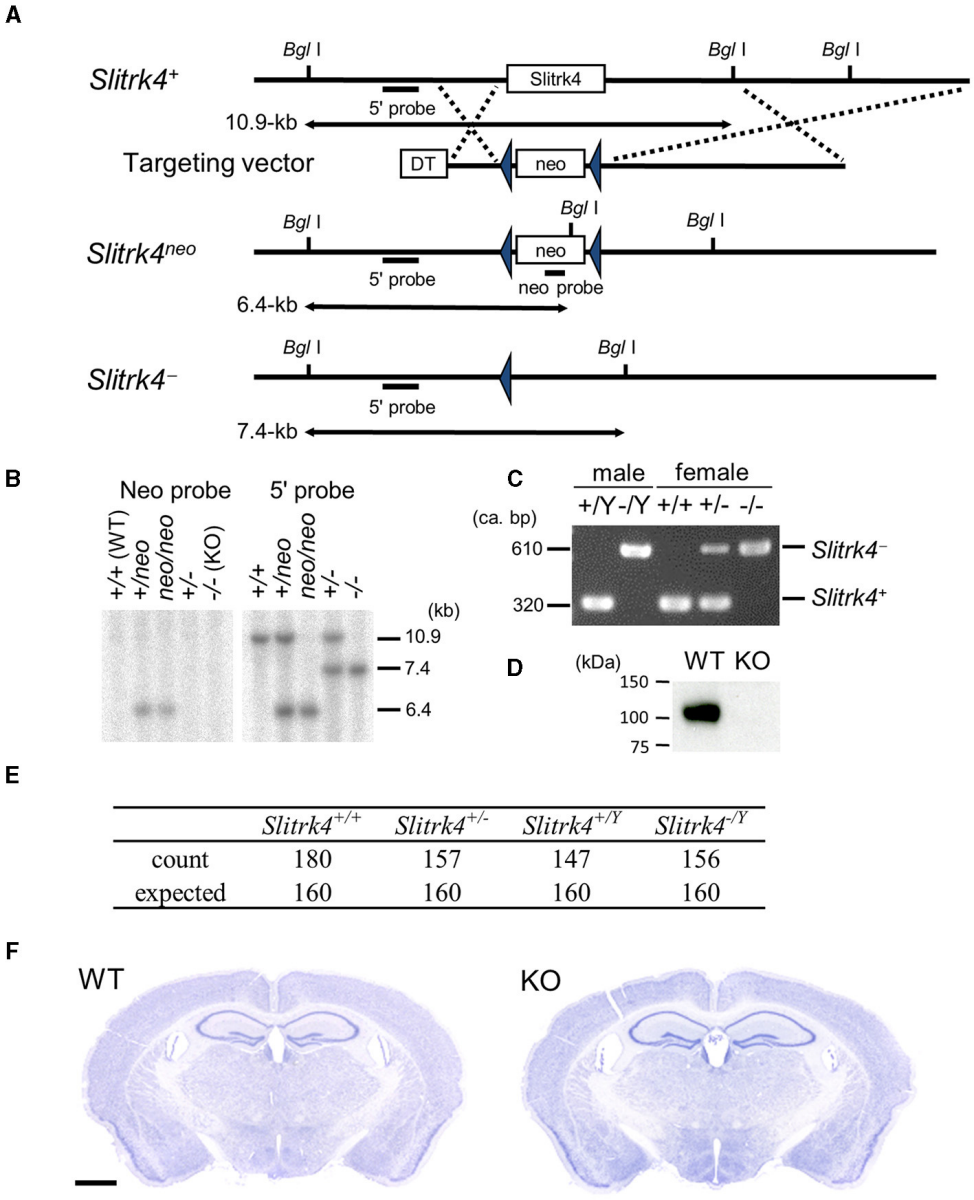
Targeted disruption of *Slitrk4* gene. **(A)** Structure of wild-type *Slitrk4* gene, targeting vector, and mutated alleles. Locations of the probes for Southern blotting (5′ and neo probes) are also shown. *DT*, diphtheria toxin A; *neo*, neomycin-resistance gene. **(B)** Confirmation of homologous recombination by Southern blot. **(C)** Genotyping of *Slitrk4*^+/*Y*^and *Slitrk*^−/*Y*^ for male mice, and *Slitrk4*^+/+^, *Slitrk4*^+/−^ and *Slitrk4*^−/−^ for female mice by genomic PCR. **(D)** Western blot performed on proteins prepared from hippocampus and amygdala of adult *Slitrk4*^+/*Y*^(WT) and *Slitrk4*^−/*Y*^ (KO) mice. **(E)** Summary of genotyping analysis. Genotypes of progenies 640 progenies derived from WT male (*Slitrk4*^+/*Y*^) × heterozygous female (*Slitrk4*^+/−^) mating. Genotyping was carried out at 3–4 weeks-old. *P* = 0.29 in χ^2^ test. **(F)** Cresyl violet staining for adult WT and KO brain coronal sections. Scale bar, 1 mm.

*Slitrk4*-null mutant mice were generated as described (Katayama et al., 2009) (Figures 1A–C). Briefly, to construct the *Slitrk4* targeting vector, overlapping *Slitrk4* genomic clones were isolated from a phage library derived from mice of the 129SV strain (Stratagene, La Jolla, CA). The targeting construct contained 3.8-kb 5′ and 5.5-kb 3′ homology regions, and the 1.3-kb fragment containing the open reading frame of *Slitrk4* was replaced with the phosphoglycerol kinase (PGK)-neo expression cassette flanked by a *loxP* sequence. E14 ES cells were electroporated with the targeting construct and selected using G418. Drug-resistant clones were analyzed by southern blotting. *Eco*RV-digested genomic DNA was hybridized with a 1.0-kb 5′ genomic fragment corresponding to the genomic sequence outside of the targeting vector and a 0.6-kb *Pst*I PGK-neo probe, respectively. Chimeric mice were generated by injecting targeted ES cells into C57BL/6J blastocysts. To excise the PGK-neo cassette, germline-transmitted mice were first mated with *Cre recombinase* transgenic mice under the control of the cytomegalovirus immediate-early enhancer-chicken β-actin hybrid (CAG) promotor (Sakai and Miyazaki, 1997). Correct excision of the PGK-neo cassette was confirmed by southern blotting. Mice carrying the mutated *Slitrk4* allele were backcrossed to C57BL/6J for more than six generations before analysis. Genotyping of progenies was performed by southern blot or PCR analysis of DNA isolated from tail samples; the PCR primers for genotyping are as follows: Slitrk4_F, 5′-CCTTGTGCAGGGGACATTAGAAAATAAG-3′; WT_R, 5′-CATCTGTTCCACATA CTGGCTGGCT-3′; KO_R, 5′-TGATATTGCTGAAGAGCTTGGCGGCGAAT-3′.

DBA2 mice were purchased from Nihon SLC (Shizuoka, Japan).

### 2.2 Generation of anti-Slitrk4 antibody

Polyclonal anti-Slitrk4 antibody was raised in a rabbit against peptides corresponding to the cytoplasmic region of mouse Slitrk4 (CDKKNKKSLIGGNHSKIVVEQRK). Peptides were synthesized and conjugated to keyhole limpet hemocyanin through cysteine added to the N-terminus of the peptide. After immunization by conventional methods, antisera were obtained, and the antibody was purified by affinity chromatography with the immunized peptide. The specificity of the antibody was confirmed by the absence of corresponding band in the KO brain lysates (Figure 1D; Supplementary Figures 1, 2).

### 2.3 Protein extraction and western blot

Samples were taken from the whole brain region using a biopsy punch (Kai Medical). The specimens were homogenized in RIPA buffer [50 mM Tris-HCl pH 8.0, 150 mM sodium chloride, 1% NP-40, 0.5% sodium deoxycholate, 0.1% SDS, 1 mM EDTA, and complete protease inhibitor cocktail (Roche Diagnostics, Mannheim, Germany)]. Ten micrograms of the extract were loaded onto a 7.5% SDS-PAGE gel, electrophoresed, and transferred to a polyvinylidene fluoride membrane (Millipore, Billerica, MA). Rabbit polyclonal anti-Slitrk4 and mouse monoclonal anti-βIII tubulin (5G8, G7121, Promega), anti-PSD-95 (6G6-1C9, MA1-045, ThermoFisher), and anti-synaptophysin (SVP-38, S9788, Sigma) antibodies were used as primary antibodies. After incubation with appropriate secondary antibodies conjugated to horseradish peroxidase, the signals were detected using an ECL Plus kit (GE Healthcare, Buckinghamshire, UK).

### 2.4 Subcellular fractionation

Adult mice were dissected and homogenized in ice-cold homogenization buffer (0.32 M sucrose, 1 mM NaHCO_3_, 1 mM MgCl_2_, 0.5 mM CaCl_2_, 2 μg/mL aprotinin, 2 μg/mL leupeptin, 1 μg/mL pepstatin) in a Teflon-glass homogenizer. For subcellular fractionation, homogenates were fractionated by differential centrifugation, as previously describe (Carlin et al., 1980; Morimura et al., 2017). Postsynaptic density fractionation of the adult mouse brain was performed as previously described (Fallon et al., 2002). The protein concentration was quantified using a BCA kit (Pierce, Rockford, IL, USA).

### 2.5 Hippocampal neuron culture, transfection, and immunostaining

Culture and immunostaining of hippocampal neurons were performed as described in a previous study (Morimura et al., 2017). Hippocampal neurons prepared from embryonic day 18 rat embryos were transfected with pCAG-Myc:Slitrk4-ires-GFP or its empty vector plasmid at 8 days *in vitro* using Lipofectamine2000 (ThermoFisher) and were fixed at 23 days *in vitro*. Myc:Slitrk4 was designed to express mouse Slitrk4 protein with Myc epitope tag at the NH2 terminus of extracellular domain after cleavage of endogenous signal peptide sequence. For primary antibodies, we used antibodies against VGAT (1:200, AB5062P, Millipore), antibodies against PSD-95 (1:100, K28/43, NeuroMab), gephyrin (1:250, 147 011, Synaptic Systems), synaptophysin (1:250, SVP-38, Sigma), and Myc (1:500, A14, Santa Cruz). The bound antibodies were detected with Alexa Fluor-conjugated secondary antibodies (1:2,000, Jackson ImmunoResearch).

### 2.6 Behavioral analysis

#### 2.6.1 Home cage activity

Spontaneous activity of mice in their home cage was measured using the 24-channel ABsystem 4.0 (Neuroscience, Tokyo, Japan). Cages were set individually into stainless steel compartments of a negative breeding rack (JCL, Tokyo, Japan). An infrared sensor was placed on the ceiling of each compartment to detect mouse movements 5 times *per sec*. Home cage activity was measured for 1 week from 16:00 on day 1 until 16:00 on day 8.

#### 2.6.2 Open field test

Each mouse was placed in the center of an open field apparatus [50 × 50 × 40 (H) cm] illuminated by light-emitting diodes (LEDs; 70 lux at the center of the field) and then allowed to move freely for 15 min. Distance traveled (cm) and duration (%) in the center area of the field (30% of the field) were adopted as the indices, and the relevant data were collected every 1 min. Data were collected and analyzed using Image J OF4 (O'Hara, Tokyo, Japan).

#### 2.6.3 Elevated plus maze test

A single channel of the elevated plus maze [closed arms: 25 × 5 × 15 cm (H); open arms 25 × 5 × 0.3 cm (H)] was equipped in the same sound-proof room as the open field and the light-dark (LD) box. The floor of each arm was made of white plastic, and the walls of the closed arms and the ridge of the open arms were made of clear plastic. Closed arms and open arms were arranged orthogonally 60 cm above the floor. The light condition was 70 lux at the center platform of the maze (5 × 5 cm). In the elevated plus maze test, mice were individually placed on the center platform facing an open arm, and the mice were allowed to move freely in the maze for 5 min. The total distance traveled, % time spent in the open arms, and percentage of open arm entries were measured as indices. Data were collected and analyzed using Image J EPM (O'Hara, Tokyo, Japan).

#### 2.6.4 Light-dark box test

A four-channel LD box system was equipped in the same sound-proof room as the open field. Each light box was made of white plastic [20 × 20 × 20 (H) cm] and illuminated by LEDs (250 lux at the center of the box), a CCD camera was equipped on the ceiling, and each dark box was made of black plastic [20 × 20 × 20 (H) cm] and an infrared camera was installed on the ceiling. There was a tunnel for transition on the center panel between the light box and dark box (3 × 5 cm) with a sliding door. In the LD box test, mice were individually introduced into the light box, and the door of the tunnel was automatically opened 3 s after the introduction of the mouse. The mice were allowed to move freely in the LD box for 10 min. The total distance traveled, % distance traveled in the light box, % duration spent in the light box, number of transitions between light and dark box, and first latency to enter the dark box were measured as indices. Data were collected and analyzed using ImageJ LD4 (O'Hara, Tokyo, Japan).

#### 2.6.5 Fear conditioning

Classical fear conditioning test consisted of three parts: a conditioning trial (day 1), a context test trial (day 2), and a cued test trial (day 3). Fear conditioning was performed in a clear plastic chamber equipped with a stainless-steel grid floor (34 × 26 × 30 cm). A CCD camera was installed on the ceiling of the chamber and connected to a video monitor and computer. The grid floor is wired to a shock generator. White noise (65 dB) was supplied by a loudspeaker as an auditory cue (CS). The conditioning trial consisted of a 2 min exploration period followed by two CS-US pairings separated by 1 min. A US (foot shock: 0.5 mA, 2 s) was administered at the end of the 30 s CS period. A context test was performed in the same conditioning chamber for three min in the absence of white noise 24 h after the conditioning trial. Furthermore, a cued test was performed in an alternative context with distinct cues; the test chamber was different from the conditioning chamber in brightness (almost 0–1 lux), color (white), floor structure (no grid), and shape (triangular). The cued test was conducted 24-h after the contextual test was finished and consisted of a 2-min exploration period (no CS) to evaluate the nonspecific contextual fear followed by a 2-min CS period (no foot shock) to evaluate the acquired cued fear. The rate of freezing response (immobility excluding respiration and heartbeat) of mice was measured as an index of fear memory. Data were collected and analyzed using ImageJ FZ2 (O'Hara, Tokyo, Japan).

#### 2.6.6 Social interaction test

Subject mice were individually placed in the center of a white open field apparatus (40 × 40 × 30 cm). Immediately after the introduction of the subject mouse, a target mouse (8–9-weeks-old C57BL/6J male mice, a naïve mouse was used for each subject mouse) was also introduced into the same open field. The duration of contact behavior was measured for 6 h. Contact or separation of mice was expressed “1” if two mice contacted or “2” if separated. Data were collected and analyzed using a personal computer and commercially available software (Time HC: O'Hara, Tokyo, Japan).

#### 2.6.7 Resident-intruder test

Male WT and Slitrk4 KO mice (residents) were housed individually for 4 weeks, and 4- weeks-old male DBA2 mice (intruders) were housed in groups. The home cages of the residents did not change for at least 3 days before testing. The experiment was performed under dim illumination (7 lux) with constant fan noise (60 dB). All mice were acclimated to the conditions for at least 30 min before testing. The test was started by placing an unfamiliar intruder in the home cage of a singly housed resident, and their behaviors were recorded on video for 10 min. Social responses observed in the resident were scored by the counts and the time spent in contact (including approach) to the intruder mouse, hiding (hiding the head or body under bedding material), attacking (biting with immediate escape response of the intruder), and standing. The scoring for each behavior was performed by an observer who was trained and blinded to the genotype.

#### 2.6.8 Social discrimination test

The test was performed as described in a previous study (Takashima et al., 2011) using the OF test apparatus with a luminance of 70 lux. The test consisted of a habituation session, the first test session, and a second test session. Each session lasted for 10 min. In the first test session, a mouse (7-week-old male DBA2 mice new to the test mouse was placed in one of two cylindrical cages. In the second test session, another mouse, which was also new to the test mouse, was placed in the remaining cylindrical cage. The times spent in the two corner squares containing the cylinders within the 3 × 3 square subdivision (17.7 × 17.7 cm square) were measured with Image J OF4 (O'Hara).

#### 2.6.9 Hidden cookie test

On day 1, 8-months-old male mice were fed a butter cookie for 24 h. On day 2, mice were deprived of food for 24 h. On day 3, mice were moved to a new cage [17 × 28 × 12 cm (H)] with thick (ca. 5 cm) pulp chips (TEK-Fresh, ENVIGO) and habituated for 1 h. After a piece of the cookie was placed at the bottom of the bedding material. The mice were placed again on the top of the bedding materials, and the latencies to reach, dig the cookie-buried area, and eat the cookie were measured.

#### 2.6.10 Tail suspension test

Mice were attached to a wire by using an adhesive tape placed ~1.5 cm from the tip of the tail and suspended 30 cm above the floor. The duration of immobility was recorded for 5 min.

#### 2.6.11 Forced swimming test

Each mouse was placed in a glass cylinder (30-cm high, 10-cm diameter) containing 20 cm of water maintained at 23–25°C. The duration of immobility was recorded for 5 min.

#### 2.6.12 Acoustic startle response and prepulse inhibition

Mice were habituated in their home cages for 1 h to 65-dB white noise. They were then placed in standard startle chambers. Each session was initiated with a 5-min acclimation period of white noise at 65 dB, followed by 10 successive 120-dB tones to elicit the startle response (40 ms). Nine different trial types were then presented: 70, 75, 80, 85, 90, 95, 100, 110, or 120 dB (40 ms) with a background noise of 65 dB. Each trial was presented five times, and the average response on each trial was calculated. Immediately after the startle response trial, the PPI session was initiated. During each PPI session, the mouse was exposed to the following types of trials: omission of stimuli (no-stimulus trial), a startle-alone trial (120 dB), and three prepulse combinations (prepulse-pulse trials) using three prepulse intensities: 70, 75, and 80 dB. Each PPI session consisted of 10 presentations for each trial. PPI was assessed for each animal as a percentage (%PPI): {1 – (mean startle to prepulse-pulse trial)/(mean startle to pulse only trial)} × 100. The apparatus and software used for data analysis were commercially available (Mouse Startle; O'Hara).

### 2.7 Auditory brainstem response

To measure ABRs, mice were anesthetized with an intraperitoneal injection of 60 mg/kg sodium pentobarbital (Nembutal, Dainippon Pharmaceutical Co., Ltd., Osaka, Japan), and needle electrodes were inserted at the vertex and pinna with a ground near the tail. ABRs were evoked with 4-ms tone pips at 40 per second with a 0.4-ms cosine square rise-fall envelope and alternating polarity to remove frequency-following responses. The voltage difference between the pinna and vertex was amplified (10,000×), filtered, digitized at 100 kHz, and averaged across 512 presentations. The sound level was decreased in 10-dB steps from an 80-dB sound pressure level. The threshold, amplitude, and latency of the responses were defined by visual inspection of the stacked waveforms.

### 2.8 Electrophysiology

Coronal amygdaloid slices (400-μm thickness) containing the LA were prepared from 8- to 12-week-old male *Slitrk4*^−/*Y*^ mice and littermate controls (*Slitrk4*^+/+^) and placed in a humidified interface-type holding chamber for at least 1 h before recordings, using standard procedures (Miwa et al., 2008). Slices were perfused with a medium that was saturated with 95% O_2_ and 5% CO_2_ and contained (in mM) 119 NaCl, 2.5 KCl, 2.5 CaCl_2_, 1.3 MgSO_4_, 1.0 NaH_2_PO_4_, 26.2 NaHCO_3_, and 11 glucose. Whole-cell patch-clamp recordings were made from principal neurons in the dorsal subdivision of the LA with a MultiClamp 700B patch-clamp amplifier (Molecular Devices, Union City, CA, USA). The pipette solution contained (in mM): 135 KMeSO_4_, 10 HEPES, 0.2 EGTA, 8 NaCl, 2 Mg-ATP, and 0.3 Na_3_-GTP (pH 7.2; 290–310 mOsm) for current-clamp recordings; 122.5 cesium gluconate, 17.5 CsCl, 10 HEPES, 0.2 EGTA, 8 NaCl, 2 Mg-ATP, and 0.3 Na_3_-GTP (pH 7.2; 290–310 mOsm) for voltage-clamp recordings. The recording electrodes had resistances of 3–7 MΩ. The series resistance was 10–30 MΩ and was monitored online throughout the experiment. The experiments were rejected if the series resistance changed by more than 20%. The signal was filtered at 5 kHz and digitized at 20 kHz with pClamp9.2 software (Molecular Devices). For current-clamp experiments, cells were clamped at −80 mV with DC current injection. For evoking synaptic responses, a bipolar stimulating electrode placed in the ventral striatum just medial to the LA to stimulate fibers originating in the auditory thalamus and was stimulated at 0.1 Hz and the stimulus strength was adjusted to evoke excitatory postsynaptic potentials (EPSPs) with amplitudes of 3–5 mV. Long-term potentiation (LTP) was induced by the theta-burst pairing protocol: trains of four stimulus at 100 Hz were paired with intracellular current injection (1 nA, 2 ms) at 10-ms intervals. A train of ten of such pairings was made at 5 Hz, and this train was repeated four times at 10 s intervals. For feedback inhibition experiment, cells were clamped at −60 mV with DC current injection and then injected with four current pulses (1 nA, 2 ms) at 10-ms intervals. The feedback inhibition amplitude was quantified with the difference in the membrane potentials between pre-current pulses membrane potential and the post-current pulses membrane potential. For feedforward inhibition experiment, at a membrane potential of −60 mV, low-intensity afferent stimulation elicited a biphasic synaptic response in principal neurons, consisting of a short-latency EPSP followed by an inhibitory postsynaptic potential (IPSP). The feedforward inhibition amplitude was quantified with the EPSP/IPSP ratio calculated from EPSP and IPSP peak amplitudes. Miniature excitatory postsynaptic currents (mEPSCs) were recorded at −80 mV in the presence of 100 μM picrotoxin and 1 μM tetrodotoxin (TTX). Miniature inhibitory postsynaptic currents (mIPSCs) were recorded at 0 mV in the presence of 10 μM 6-cyano-7-nitroquinoxaline-2,3-dione (CNQX), D-(–)-2-amino-5-phosphonovaleric acid (D-APV) (50 μM), and 1 μM TTX. Experiments were carried out in a genotype-blinded manner. The amplitude and frequency of miniature events were analyzed with the AxoGraph X 1.6.3 (Axograph Scientific, Australia), which uses a detection algorithm based on a sliding template optimally scaled to fit the events at each position.

### 2.9 Immunostaining

Identical conditions were used for immunostaining, as well as capture and analysis of confocal images for each condition. Each experiment was performed blinded. Mice were anesthetized with isoflurane and perfused with 4% paraformaldehyde in 0.1 M phosphate buffer (pH 7.4), post-fixed, and cryoprotected in 30% (w/v) sucrose in PBS. Cryostat sections (10 or 12 μm) were incubated in blocking solution containing PBS, 2 or 5% normal goat serum, and 0.1% Triton X-100, then overnight at 4°C with VGAT (1:200, AB5062P, Millipore), antibody to VGLUT1 (1:1,000, AB5905, Millipore), VGLUT2 (1:1,000, AB2251, Millipore), NR2B (1:100, N59/36, NeuroMab), synaptophysin (1:100, Ab-4, ThermoScientific), PSD-95 (1:100, K28/43, NeuroMab), calbindin (1:200, AB1778, Millipore), calretinin (1:200, AB149, Millipore), calretinin (1:1,000, 214 104, Synaptic Systems), parvalbumin (1:200, AB15736, Millipore), SST (1:200, MAB354, Millipore), or activated caspase (1:400, 9661, Cell Signaling) in blocking solution, then with Alexa Fluor-conjugated secondary antibodies (1:2,000, Jackson ImmunoResearch). Confocal images were captured sequentially on a Fluoview FV1000 confocal system from five separate fields per anatomical region per animal. Four wild-type and five knockout animals were used for the analysis (Figure 6). Images were analyzed using the ImageJ software. A single threshold was set for each staining condition to capture clusters that were clearly distinguishable and to minimize the merged clusters. The number, size, and intensity of puncta were measured. For the interneuron counting in Figure 7, the jointed images for entire brain sections were obtained using a Keyence BZ-X700 microscope with a 10× objective lens, and the counting was performed by naked eye observations of persons who were blinded to the genotype. Four to six coronal sections [AP, −1.34 to −2.06 mm from the Bregma sections (Paxinos and Franklin, 2001)] were analyzed to obtain a mean value for a mouse.

### 2.10 mRNA level analysis

For mRNA level analysis, RNA was isolated from punch biopsy tissues of LA using TRIzol Reagent (Thermo Fisher). cDNA was synthesized using SuperScript II reverse transcriptase (Thermo Fisher). Real-time RT-PCR analysis was carried out using Power SYBR Green PCR Master Mix (Thermo Fisher) and ABI PRISM 7900HT (Thermo Fisher). The primer sequences are listed in Supplementary Table 1.

### 2.11 *In situ* hybridization analysis

*In situ* hybridization was performed as previously described (Aruga and Mikoshiba, 2003). For the double labeling analysis, digoxigenin-labeled Slitrk4 probe, fluorescein-labeled calretinin probes, alkaline phosphatase-conjugated anti-digoxigenin antibody, and peroxidase-conjugated anti-fluorescein antibody were used for the analysis. Peroxidase-derived signals were detected using a Cy3/Cy5 TSA Plus kit (PerkinElmer).

### 2.12 Induction of GABAergic neurons from ES cells

E14 ES cells derived from 129P2/OlaHsd or its derivative, Slitrk4-lacking ES cells, were subjected to *in vitro* differentiation into GABAergic cortical interneuron-like cells. Induction was performed according to Tischfield and Anderson (2017). Briefly, floating ES cells were cultured in KSR:N2 (1:1) medium containing Wnt inhibitor (10 μM XAV-939) and BMP inhibitor (250 nM LDN-193180) for 3 days to form embryoid bodies. The embryoid bodies were then dissociated with trypsin-EDTA (0.05% trypsin) and plated onto poly-L-lysine and laminin-coated dishes with the same culture medium. Two days later (Day 5), the culture medium was replaced with KSR:N2 (1:1) containing 10 ng/mL FGF-2 and 20 ng/mL IGF-1 with or without 1 μM purmorphamine (Smoothened agonist). Three days later (day 8), the cells were replated and kept in N2/KSR (1:1) containing FGF-2 (10 ng/mL), IGF-1 (20 ng/mL), and 10-μM Y-27632 (ROCK inhibitor) with or without 1-μM purmorphamine. The cultured cells were subjected to qPCR analysis and immunoblot analyses on days 16 or 17.

### 2.13 Statistics

Data are presented as means ± standard deviation (SD) unless otherwise stated. The sample sizes for each experiment were determined such that the power and significance in the two-sided test were 80 and 5%, respectively (Festing, 2018). However, the number of samples from the animals was minimized empirically. The Student's *t*-test, Welch's *t*-test, or Mann-Whitney *U*-test was used to determine the statistical significance of differences between the two groups. Homogeneity of variances were tested with *f*-test. Normality of distribution was tested with Kolmogorov-Smirnov test. To examine the influence of the two independent categorical variables, a two-way analysis of variance (ANOVA), repeated-measures ANOVA was performed. Statistical Differences were considered statistically significant at *P* < 0.05. In *Result* section, *percentage values* mean (KO_mean_ – WT_mean_)/WT_mean_ × 100, *P*-values are those obtained by two-tailed unpaired Student's *t*-tests between WT and KO mice (*n* = mouse number) unless otherwise stated.

## 3 Results

### 3.1 Slitrk4 protein localization

**Figure 2:**
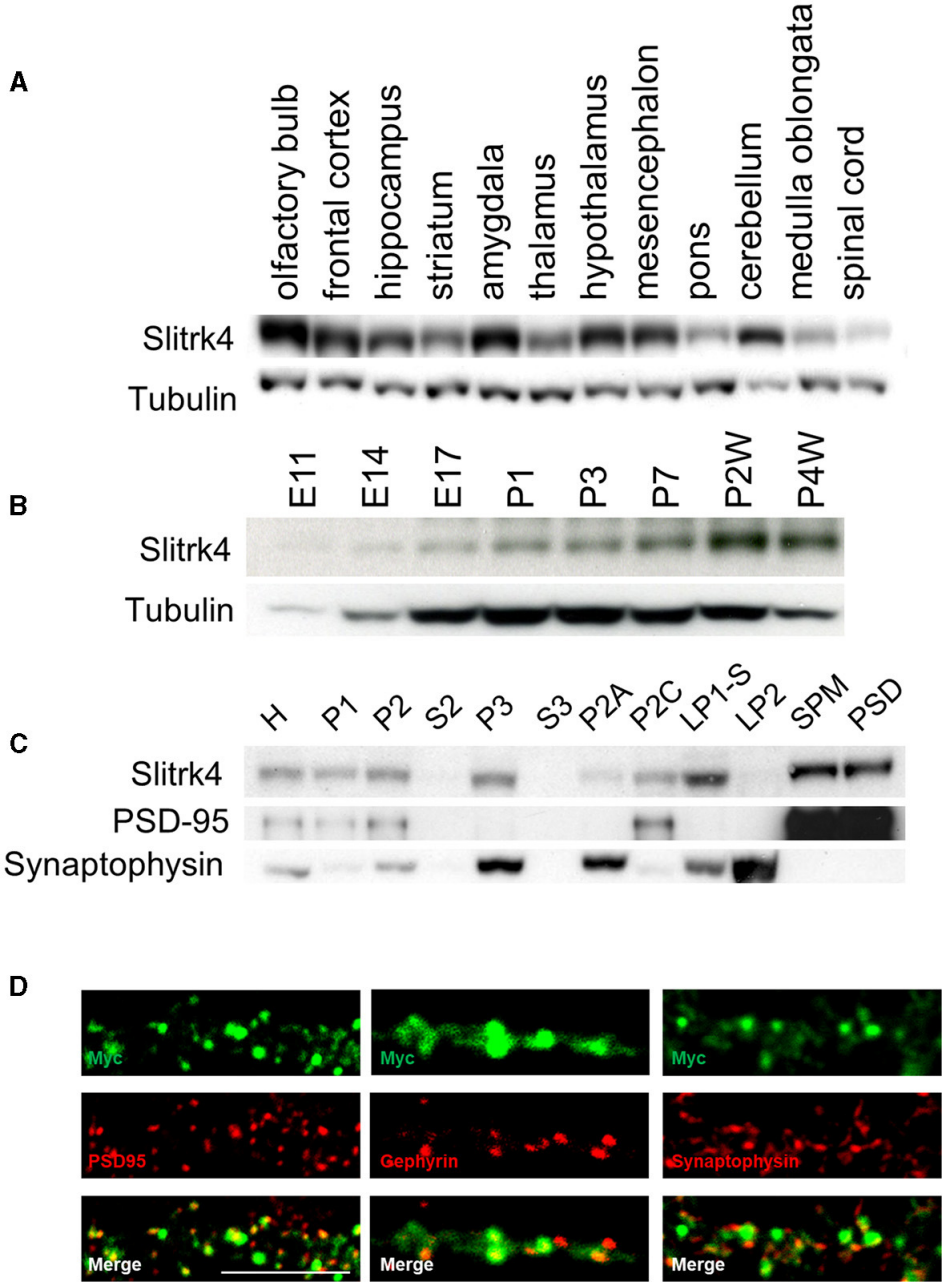
Distribution of Slitrk4 protein. **(A)** Regional distribution in the central nervous system of mice. **(B)** Temporal profile during development. **(C)** Subcellular distribution. The mouse brain lysates are biochemically fractionated. **(A–C)** Immunoblot using antibodies against Slitrk4, tubulin, PSD95, or synaptophysin. Five micrograms of protein are electrophoresed in each lane. E, embryonic day; P, postnatal day; P2A, myelin; P2C, mitochondria; LP1-S, Triton-soluble membrane fraction of synaptosome; SPM, synaptosomal plasma membrane; LP2, crude synaptic vesicle; PSD, postsynaptic density. **(D)** Immunostaining showing localization of Myc-Slitrk4 (green), PSD95 (red, an excitatory postsynaptic protein), gephyrin (red, an inhibitory postsynaptic protein), and synaptophysin (red, presynaptic protein) in cultured rat hippocampal neurons. Scale bar, 10 μm.

In the organs of adult mice, Slitrk4 mRNA is predominantly detected in the brain (Aruga and Mikoshiba, 2003). We first examined the distribution of Slitrk4 protein in the brains of adult male mice by immunoblot analysis using an anti-Slitrk4 antibody. Slitrk4 was detected in all examined regions of the central nervous system and was abundant in the olfactory bulb and amygdala (Figure 2A; Supplementary Figures 1, 2). In the olfactory bulb, Slitrk4 mRNA was detected strongly in the anterior olfactory nuclei (Allen Brain Atlas, http://mouse.brain-map.org/experiment/show/74882938) (Aruga and Mikoshiba, 2003). During brain development, the expression increased from embryonic day 14 to 2 weeks after birth in total brain lysates (Figure 2B). In subcellular fractions of the brain lysates, Slitrk4 was enriched in the synaptosomal plasma membrane and postsynaptic density fractions (Figure 2C). Transfected Myc-Slitrk4 in hippocampal neurons overlapped well with the excitatory postsynaptic marker PSD-95 (45/45) and rarely with the inhibitory postsynaptic marker gephyrin (2/32), or with the presynapse marker synaptophysin (3/45) (Figure 2D). These results collectively indicate that the Slitrk4 protein is preferentially distributed in excitatory postsynapses in adult mouse brains.

### 3.2 Generation of Slitrk4-deficient mice

To clarify the role of *Slitrk4 in vivo*, we generated *Slitrk4*-deficient mice. In the *Slitrk4* knockout allele (*Slitrk4*^−^), the open reading frame of Slitrk4 was replaced with a loxP sequence (Figures 1A–C). *Slitrk4*^−/*Y*^ brain lysate lacked the Slitrk4 protein (Figure 1D). In male wild-type (WT, *Slitrk4*^+/*Y*^) × female heterozygote (*Slitrk4*^+/−^) mating, *Slitrk4*^−/*Y*^ mice were born and developed in the expected Mendelian ratio (Figure 1E). Both males (*Slitrk4*^−/*Y*^) and females (*Slitrk4*^−/−^) were fertile. In this study male mice were used to avoid effects of estrous cycles on behavioral phenotypes in females (Meziane et al., 2007). Hereafter, we refer to *Slitrk4*^−/*Y*^ as “Slitrk4 KO” or “KO” for simplicity. Body weight was comparable between WT and KO males (WT, 26.0 ± 1.42 g; KO, 25.4 ± 1.22 g; at 13–14 weeks-old, *n* = 20 per genotype). KO brains did not show any obvious abnormalities in the gross histological architecture (Figure 1F).

### 3.3 Behavioral abnormalities in Slitrk4 KO mice

We carried out a set of behavioral experiments (Table 1) to assess the brain function of Slitrk4 KO mice. Behavioral tests were performed using adult male KO and WT littermates. The results are summarized as follows.

**Table 1:** Summary of Slitrk4 KO behavioral phenotypes.

**Test**	**Index**	**Change**	***p*-values**	**Stat-values**	**Stat. test**	**WT n**	**KO n**	**Cohort-order**
Homecage activity	Whole day	n.s.			Student	10	10	A-1
	Light phase	n.s.			Student			
	Dark phase	n.s.			Student			
Open field	Total distance	KO↓	0.038	*t* = 2.2	Student	10	10	A-2
	%center time	n.s.			Student			
Light dark box	Total distance	KO↓	0.0060	*t* = 3.1	Student	10	10	A-3
	No. of transition	KO↓	0.0063	*t* = 3.1	Student			
	Lat. to transition	n.s.			Student			
	%dist(l)	n.s.			*U*-test			
	%time(l)	n.s.			*U*-test			
Elevated plus maze	Total distance	n.s.			Student	10	10	A-4
	No. of entry	n.s.			Student			
	%time open	n.s.			*U*-test			
	%no. open	n.s.			*U*-test			
Marble burying	No. of burying	n.s.			*U*-test	9	10	F-5
	Total distance	n.s.			Student			
	Rearing	KO↓	0.023	*U* = 17.5	*U*-test			
Hole board test	Total distance	n.s.			Student	10	10	B-1
	Active time	n.s.			Student			
	Head dipping (time)	n.s.			Welch			
	No. of head dipping	KO↓	0.027	*t* = 2.5	Welch			
	Rearing (time)	n.s.			Student			
	No. of rearing	n.s.			Student			
Novel object	Total distance (habit)	KO↓	0.0014	*t* = 3.8	Student	10	10	C-1
	Total distance (test)	n.s.			Student			
	%center time (habit)	n.s.			Student			
	%center time (test)	n.s.			Student			
Tail suspension	%immobility (total)	n.s.			rmANOVA	10	10	B-6
Forced swimming	%immobility (total)	n.s.			rmANOVA	10	10	B-7
	%immobility (bin1)	KO↑	0.024	*t* = 3.7	Sidak			
Rotarod	Rotation	n.s.			rmANOVA	10	10	B-3
Wire hanging	Stayed time	n.s.			Student	10	10	F-6
Hot plate	Lick	n.s.			Student	10	10	B-4
	Flinch	n.s.			Student
	Jump	n.s.			U-test
Tail flick	Escape latency	n.s.			Student	10	10	B-5
Startle response	Startle response	KO↑	*P*_(genotype × *soundlevel*)_ = 0.050	*F*_(1, 179)_ = 2.0	rmANOVA	10	10	A-5
	Initial/final	n.s.			rmANOVA			
	PPI	n.s.			rmANOVA			
Fear conditioning (1)	Conditioning (last bin)	n.s.			U-test	10	10	A-7
	Context test	n.s.			U-test			
	Cue test (preCS)	n.s.			U-test			
	Cued test (CS)	KO↑	*P*_(genotype)_ = 0.0076	*F*_(1, 79)_ = 9.0	rmANOVA			
Fear conditioning (2)	Conditioning (last bin)	n.s.	P_(genotype)_ = 0.00067	F_(1, 79)_ = 16.8	*U*-test	10	10	D-1
	Context test	n.s.			*U*-test			
	Cue test (preCS)	n.s.			*U*-test			
	Cued test (CS)	KO↑			rmANOVA			
Fear extinction	Early extinction	KO↑	*P*_(genotype)_ = 0.013	*F*_(1, 87)_ = 7.5	rmANOVA	11	11	E-1
			*P*_(genotype × *time*_*bin*)_ = 0.59	*F*_(3, 87)_ = 0.65				
			*P*_(genotype × *time*_*bin*)_ = 0.59	*F*_(3, 87)_ = 0.65				
	Late extinction	n.s.	*P*_(genotype)_ = 0.75	*F*_(1, 87)_ = 0.10	rmANOVA			
			*P*_(time_bin)_ = 0.0030	*F*_(3, 87)_ = 5.2				
			*P*_(genotype × *time*_*bin*)_ = 0.42	*F*_(3, 87)_ = 0.96				
Morris water maze	Total distance	n.s.			Student	10	10	A-6
	No movement time	n.s.			Student			
	Latency	n.s.			Student			
	%time in target quadrant	n.s.			*U*-test			
	%target crossing count	n.s.			*U*-test			
Social interaction	No. of particle	KO↑	*P*_(genotype × *timebin*)_ = 0.00036	*F*_(1, 119)_ = 5.1	rmANOVA	10	10	B-2
Resident intruder	Bite	KO↓	0.031	*t* = 2.5	Welch	11	11	G-1
	Contact	n.s.			Student			
	Hide	KO↑	0.0020	*t* = 4.5	Student			
	Stand	n.s.			Student			
Social discrimination	Total distance (habit.)	KO↓	0.00024	*t* = 4.6	Student	10	10	C-2
	Total distance (test1)	KO↓	0.044	*t* = 2.2	Student			
	Total distance (test2)	KO↓	0.041	*t* = 2.2	Student			
	Cage approach	n.s.			Student			
Buried food-seeking	Digging latency	n.s.				5	5	H-2
	Eating latency	KO↓	0.0031	*t* = 5.7	Welch			

n.s., not significant differences; KO↓, KO mean was lower than WT mean; KO↑, KO mean was higher than WT mean; rmANOVA, repeated measures 2-way ANOVA; Sidak, Sidak's *post-hoc* test; Student, Student's unpaired two-tailed *t*-test; *U*-test, Mann-Whitney's *U*-test; Welch, Welch's two-tailed *t*-test; A–G, independent animal cohort group (following numerals indicate the order of behavioral test). A–F, 2.9–4.8 months-old during the test period; G and H, 8–9 months-old at the testing.

**Figure 3:**
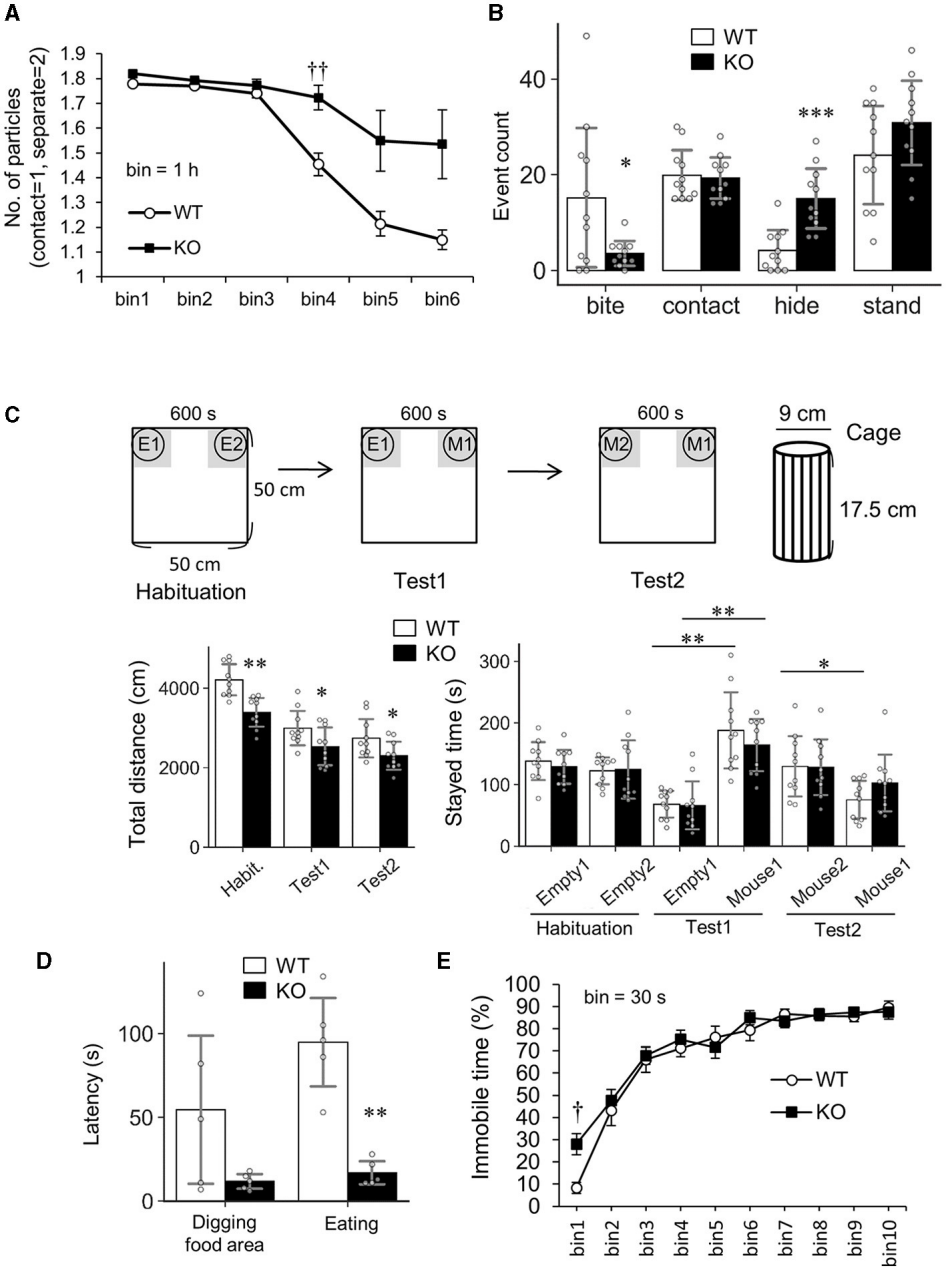
Altered social behavior and odorant perception in *Slitrk4* KO mice. **(A)** Social interaction test in an open field box. Time bin = 1 h. Values indicate the mean number of particles where separated mice and contacting mice are counted as two and one, respectively. Error bar, standard error of the mean (SEM). **(B)** Resident-intruder test. Behavior elements of resident mice (*Slitrk4* WT or KO) exposed to a younger intruder mouse are categorized into biting, contacting, hiding, or standing. The numbers of behaviors in the 10-min testing period are measured. WT, *n* = 11; KO, *n* = 11. Error bar, SD. **(C)** Social discrimination test. Total traveled distance in the entire field (*left graph*) and gray area-staying time (*right graph*) are measured. E1, Empty cage 1; E2, Empty cage 2; M1, cage with Mouse 1; M2, cage with Mouse 2. At Test2, M1 is a familiar mouse that was met during Test 1, whereas M2 is an unfamiliar mouse met for the first time at this stage. WT, *n* = 10; KO, *n* = 10. Error bar, SD. **(D)** Hidden cookie test. Latency to dig the bedding materials in the food area and latency to eat the food are measured (*left*). WT, *n* = 5; KO, *n* = 5. Error bar, SD. **(E)** Forced swimming test. Time bin = 30 s. WT, *n* = 10; KO, *n* = 10. Error bar, SEM. **(A, E)** *P* < 0.05; *P* < 0.01 in *post-hoc* Sidak's test after two-way ANOVA for repeated measures (Table 1). (B-bite, D) *P < 0.05; **P < 0.01 in Welch's *t*-test. (B-hide, C) *P < 0.05; **P < 0.01; ***P < 0.001 in Student's *t*-test.

First, Slitrk4 KO mice exhibited abnormalities in social behaviors (Figures 3A–C; Table 1). Slitrk4 KO mice spent more time alone in a novel environment with an unfamiliar mouse of the same sex (male) (*P* = 0.0056 at 3–4 h, rmANOVA with *post-hoc* Sidak test) (Figure 3A; Table 1). In the resident-intruder test, Slitrk4 KO mice showed less attacking (biting) behavior (−77%, *P* = 0.031, Welch's *t*-test) and more hiding behavior (burying heads under bedding material, +259%, *P* = 0.00020) than WT mice (Figure 3B; Table 1). In a test evaluating discrimination between familiar and unfamiliar mice in cages, approach to familiar mice was larger although the difference was not statistically significant (+36%, *P* = 0.15) (Figure 3C; Table 1).

Second, Slitrk4 KO mice showed abnormalities in the buried food-seeking test. Slitrk4 KO reached cookies hidden under the bedding materials more quickly than WT (latency to eat, −74%, *P* = 0.0031, Welch's *t*-test), suggesting an elevated olfactory function (Figure 3D; Table 1).

**Figure 4:**
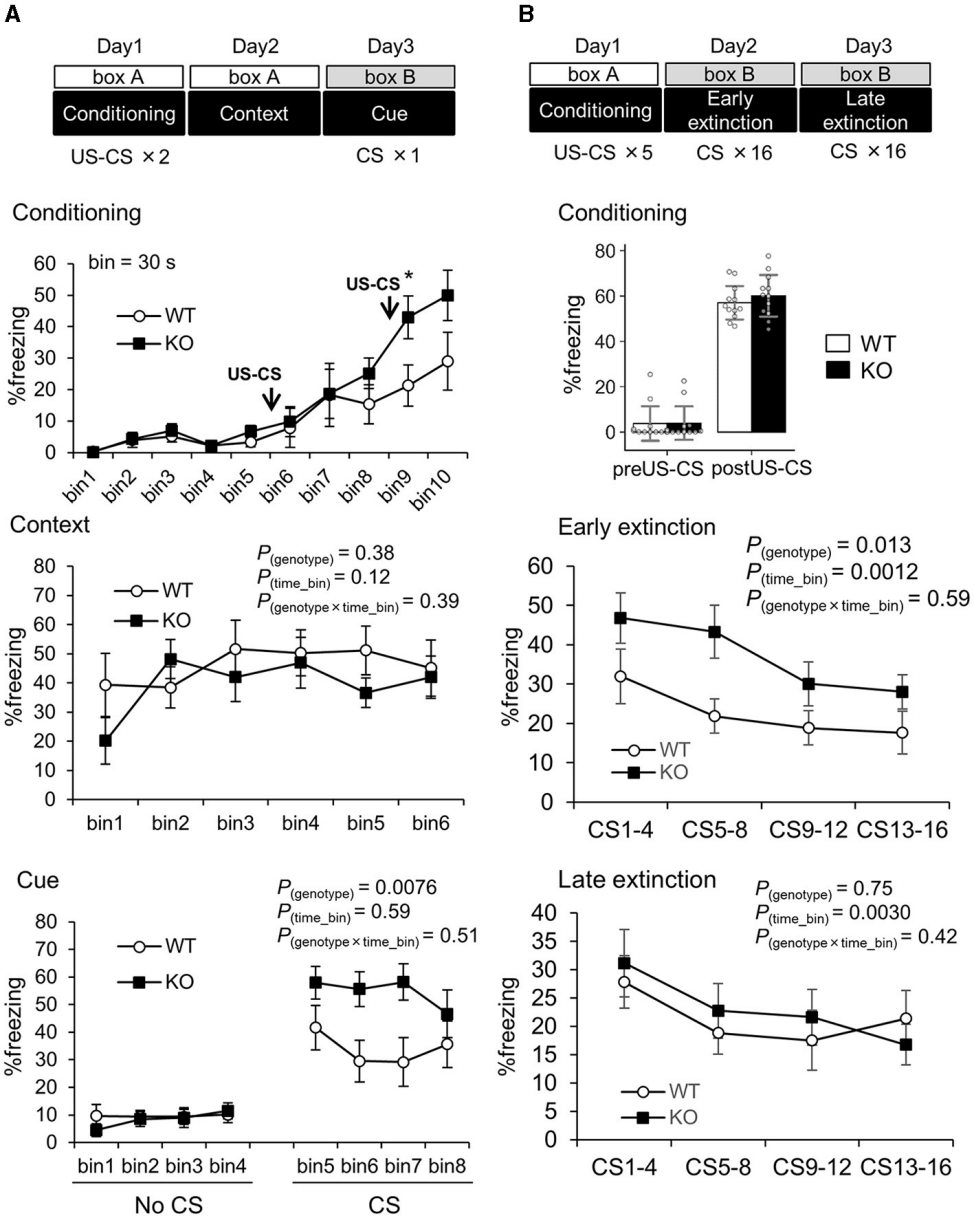
Enhanced cued fear memory in *Slitrk4* KO mice. The freezing behavior of conditioned mice is measured. **(A)** Mice were exposed to the same environmental context (box A) 24 h after two CS (30 s noise)-US (electrical footshock) paired stimuli given in box A, and to a novel environment (box B) with a CS 48 h after the conditioning. *Top*, freezing responses during fear conditioning. *Middle*, context test for fear memory. *Bottom*, cue test for fear memory. Error bar, SEM. **(B)** Extinction of experimental fear (Extinction learning). *Top*, fear conditioning including five CS-US paired stimuli were given on the day 1 in box A. Freezing before (preUS-CS) and after (postUS-CS) the conditioning was quantified. *Middle*, on the day 2, 16 CS were given during 25 min in box B (Early extinction). *Bottom*, on the day 3, 16 CS were given during 25 min in box B (Late extinction). In each assay, freezing percentage was measured for 30 s just after CS. Averages of 4 time-bins are indicated. Time bin = 30 s. WT, *n* =10; KO, *n* = 10. Error bar, SEM (line graphs), SD (bar graph). *P < 0.05, *U*-test. *P*-values indicate those in two-way ANOVA for repeated measures (genotype × time bin).

Third, fear memory function was enhanced in Slitrk4 mice. We assessed fear memory function using the classical fear conditioning test (Figure 4A; Table 1). There were no clear differences in freezing responses between WT and KO mice during auditory stimuli [white noise, conditioned stimulus (CS)]-noxious stimulus [electric foot shock, unconditioned stimulus (US)]-coupled conditioning (day 1) and replacing in the same box as the contextual test (day 2), except for that the freezing of KO was higher than that of WT in a time bin (30 s) after the second CS-US stimulus (bin 9, +102%, *P* = 0.045, *U*-test). However, in the cued test (day 3), the freezing response of Slitrk4 KO mice was higher than that of WT mice after exposure to CS [+61%, *F*_(1, 79)_ = 9.0, *P*_*genotype*_ = 0.0076; in two-way ANOVA for repeated measures, genotype and time bin as main factors]. The enhanced freezing response of Slitrk4 KO mice in the cue test was reproducible in another group of mice [+110%, *F*_(1, 79)_ = 16.8, *P*_*genotype*_ = 0.00067] (Table 1). We then examined the fear memory extinction profile. Naïve WT and Slitrk4 KO mice were conditioned as described above on the first day and 16 CSs without repeating US on the second and third days (Table 1; Figure 4B). As a result, KO freezing responses were higher than WT on the second day (early extinction) [+64%, *F*_(1, 87)_ = 7.5, *P*_*genotype*_ = 0.013 in two-way ANOVA for repeated measures, genotype and time bin as main factors], but was comparable to WT during the 17–32 successive CSs on the third day (+7.8%, late extinction, *P*_*genotype*_ = 0.75) (Table 1; Figure 4B). There were no genotype-specific effects on the extinction profiles either on the early or late extinction (early extinction, *P*_*genotype*×*time*_*bin*_= 0.59; late extinction, *P*_*genotype*×*time*_*bin*_= 0.42). These results suggested that the extinction learning was preserved in Slitrk4 KO mice. Taken together, fear memory acquisition was thought to be enhanced in the Slitrk4 KO mice.

In addition to the above three abnormalities, Slitrk4 KO locomotor activities were low in some novel environments. Spontaneous activities in their homecages were not different between WT and KO mice (Table 1; Supplementary Figure 3). However, the total distance traveled by *Slitrk4* KO mice was significantly less than that of the controls during the open field test (−14%, *P* = 0.038) (Table 1; Supplementary Figure 4), social discrimination test (habituation, −19%, *P* = 0.00024; test1, −15%, *P* = 0.044; test2, −16%, *P* = 0.041) (Figure 3C), and the light-dark (LD) box test (−19%, *P* = 0.0060) (Supplementary Figure 3). In addition, KO mice displayed significantly increased immobility time in both forced swimming during the initial 30 s period (+236%, *P* =0.024, rmANOVA with *post-hoc* Sidak test) (Table 1; Figure 3E). Prepulse inhibition of the auditory startle response tended to be reduced in Slitrk4 KO mice (−56%, *P* = 0.078, at 70 dB prepulse before 120 dB startle stimulus) without clear differences in auditory function as determined by the auditory brain stem response (Table 1; Supplementary Figure 5). There were no differences between WT and KO mice in anxiety-associated parameters, such as time spent in the center area of the open field test, time spent in open arms in the elevated plus maze test, and light box-staying time in the LD box test (Table 1). We did not observe any obvious abnormalities in noxious stimulus-associated behavioral tests (tail-flick test and hotplate test) or the hippocampal function-associated Morris water maze test (Table 1; Supplementary Figure 4).

Among the behavioral phenotypes of Slitrk4 KO mice, our attention was attracted by the enhanced fear-memory acquisition because little is known about the role of Slitrk family genes in the fear memory-related neural circuits. We therefore investigated the basis of the enhanced fear memory acquisition hereafter.

### 3.4 Thalamo-amygdala synaptic plasticity was altered in Slitrk4 KO

**Figure 5:**
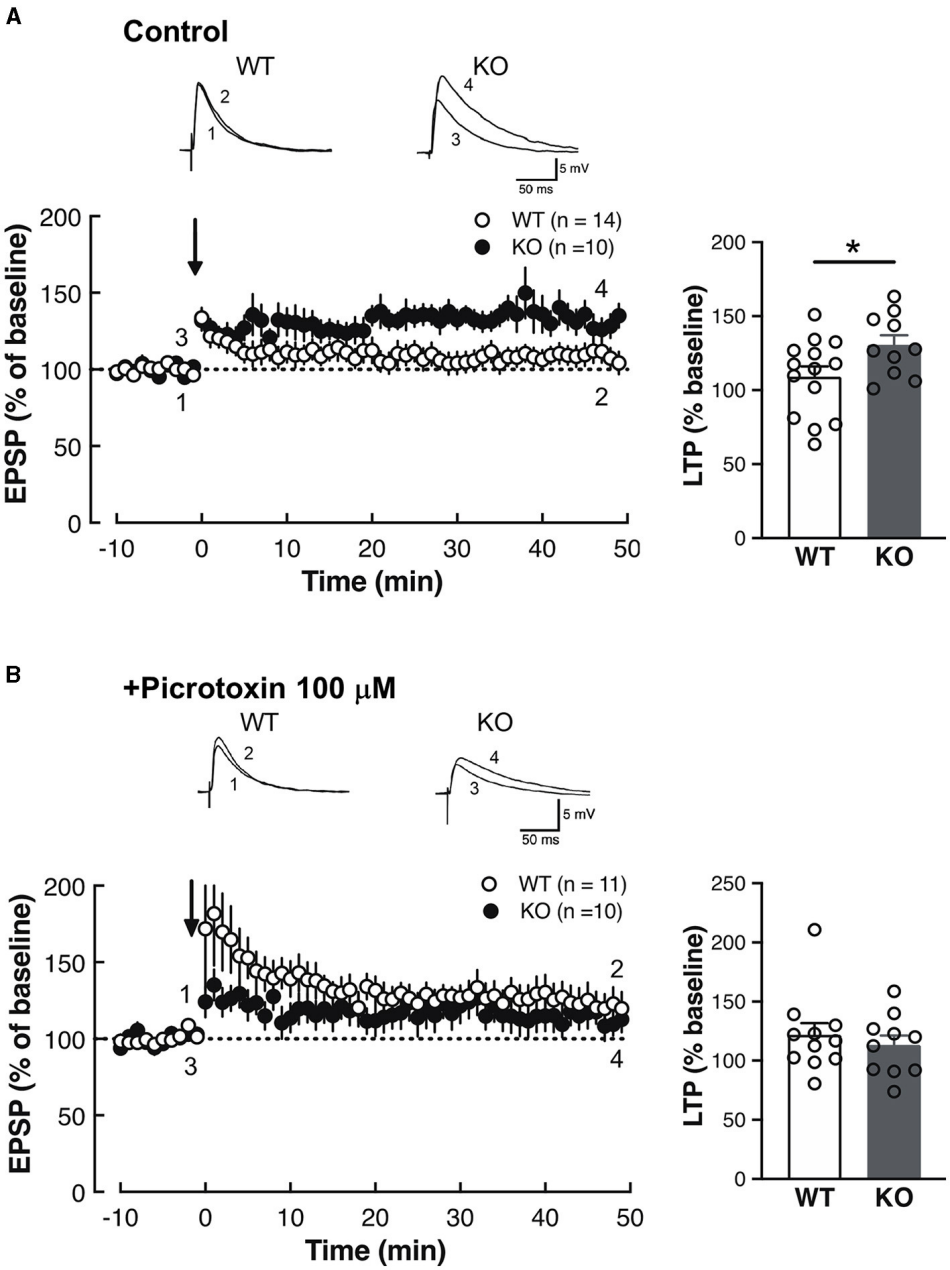
Enhanced synaptic plasticity in the LA of *Slitrk4* KO mice. **(A)** Long-term potentiation (LTP) in thalamo-amygdala afferents under normal conditions (Ringer solution). LTP of *Slitrk4* KO mice is significantly higher than that of WT. WT, *n* = 14 slices from 10 mice; KO, *n* = 19 slices from eight mice; *P = 0.044 in Student's *t*-test. **(B)** In the presence of 100-μM picrotoxin (GABA_A_ antagonist), there are no differences in LTP between genotypes. WT, *n* = 11 slices from eight mice, KO, *n* = 10 slices from 10 mice; *P* = 0.52 in Student's *t*-test. Error bar, SEM.

**Figure 6:**
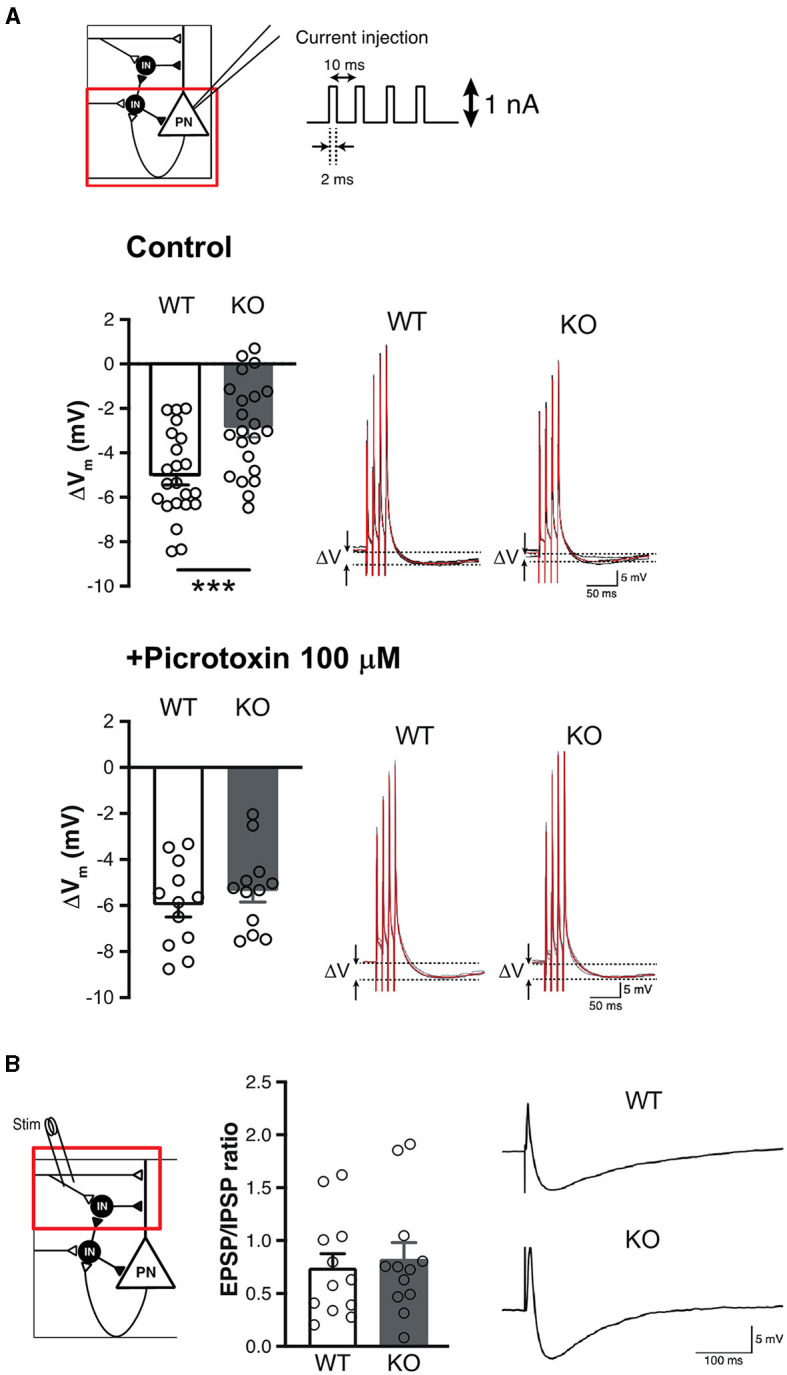
Impaired feedback inhibition in the LA of *Slitrk4* KO mice. **(A)** Measurement of feedback inhibition to pyramidal neurons in LA. Schematic view of inhibitory circuits gating LTP induction in the LA (*top, upper left*). Voltage depression (ΔV) caused by the excitation of pyramidal neurons decreased in *Slitrk4* KO mice (*top*). *n* = 22 slices from five mice per genotype; ***P = 0.00090 in Student's *t*-test. The decrement is blocked by picrotoxin (*bottom*). *n* = 12 slices from five mice per genotype; *P* = 0.41 in Student's *t*-test. **(B)** Feedforward inhibition of pyramidal neurons in LA is comparable between WT and *Slitrk4* KO mice. *n* = 12 slices from five mice for per genotype; *P* = 0.70 in Student's *t*-test. Error bar, SEM.

The above results of the fear memory cue abnormalities (Figure 4; Table 1) and Slitrk4 abundance in the amygdala (Figure 2A) led us to examine the electrophysiological properties of the thalamic auditory center (MGB) to the amygdala lateral nucleus (LA) synapse because enhanced fear memory acquisition has been correlated with altered MGB-LA synaptic plasticity in rodents (LeDoux, 2000). Whole-cell patch clamp recordings were made from the dorsal LA in coronal slices (Figures 5, 6). Long-term potentiation (LTP) was induced by the theta-burst pairing protocol (see **Materials and methods** for details). A train of ten of such pairings was made at 5 Hz, and this train was repeated four times at 10 s intervals. As a result, LTP was higher in *Slitrk4* KO mice than in WT mice (*P* = 0.044, Figure 5A) in the absence of the GABA_A_ receptor non-competitive antagonist picrotoxin but was comparable to WT (*P* = 0.52, Figure 5B) in the presence of 100 μM picrotoxin. Both the amplitude and frequency of miniature excitatory postsynaptic currents were comparable between WT and KO mice, and this was also the case for miniature inhibitory postsynaptic currents (Supplementary Figure 6). Taken together, these results indicate the possibility that MGB-LA synapse LTP was increased in *Slitrk4* KO mice in a GABAergic transmission-dependent manner without changes in the basal GABAergic synapse activity on LA (principal or excitatory) neurons.

Activity and plasticity of amygdala excitatory principal neurons are controlled by local inhibitory neurons that constitute distinct subtypes (Krabbe et al., 2018; Unal et al., 2020), including both feedback inhibition interneurons (Shumyatsky et al., 2002) and feedforward inhibition interneurons (Bissiere et al., 2003). To further investigate GABAergic dysfunction of *Slitrk4* KO mice, we tested whether feedback or feedforward type is involved in the altered plasticity in the LA of *Slitrk4* KO mice. The involvement of feedback-type interneurons was tested by examining the extent of hyperpolarization after four pulses of 100 Hz intracellular current injection into cells (1 nA, 2 ms) without synaptic stimulation (Figure 6A). There was a 5.04 mV hyperpolarization in WT but was lower in KO (2.85 mV, *P* = 0.00090). In the presence of picrotoxin, the hyperpolarization was comparable between WT and KO (WT, 5.96 mV; KO, 5.33 mV; *P* = 0.41) (Figure 6A). Feedforward-type inhibition was examined by measuring the EPSP/IPSP ratio of principal neurons under the current clamp with a holding potential of −60 mV, given a single stimulus to the input (Figure 6B). The results showed that the EPSP/IPSP ratio was not distinguishable between WT and KO mice (WT, 0.737; KO, 0.819; *P* = 0.70). These results led us to conclude that feedback-type inhibition, but not feedforward-type inhibition, in the LA was impaired in *Slitrk4* KO mice.

### 3.5 Calretinin-positive GABAergic interneuron subset was decreased in the *Slitrk4* KO amygdala

**Figure 7:**
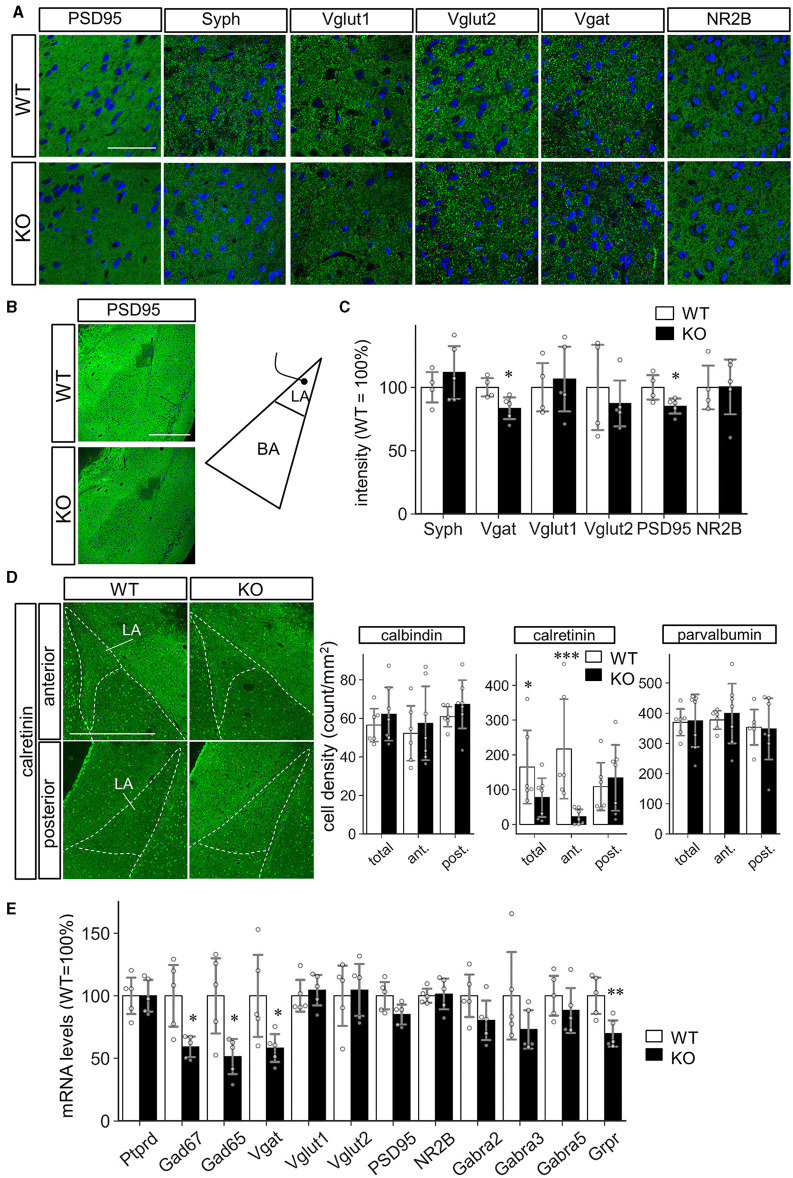
Decreased inhibitory interneurons in the LA of *Slitrk4* KO mice. **(A–C)** Synaptic markers in the LA of WT and *Slitrk4* KO mice. **(A)** Representative images of immunostaining. Scale bar, 50 μm. **(B)** Low magnification view indicating the regions for analysis (dark square regions). Scale bar, 500 μm. **(C)** Quantitative analysis of the immunopositive-particle-signal-intensity. Those for particle-signal-count and particle-signal-size are indicated in A. WT, *n* = 4 mice, KO, *n* = 5 mice. Value for a mouse is the mean of eight images. Error bar, SD.*P < 0.05 in Student's *t*-test. **(D)** The cell densities of interneuron subtypes in the LA. Immunostaining is performed using antibodies against interneuron subtype markers (calbindin, calretinin, and parvalbumin). Representative images for calretinin immunostaining (*Left*). Scale bar, 500 μm. Quantification of the cell densities of calbindin, calretinin, and parvalbumin (*Right*). Densities of the immunopositive cells in the anterior LA (Bregma −1.70 mm) and posterior LA (Bregma −2.18). WT, *n* = 6 mice; KO, *n* = 7 mice. Value for a mouse is the mean of five or six images. Error bar, SD. *P < 0.05; ***P < 0.001 in Student's *t*-test. **(E)** mRNA levels of the selected markers. Quantitative PCR analysis is carried out on cDNAs prepared from LA tissue punches. *n* = 5 mice for each genotype. Error bar, SD. *P < 0.05; **P < 0.01 in Student's *t*-test.

We then performed molecular marker analyses to clarify the basis of altered LA synaptic function in the amygdala. First, common synaptic markers (PSD95, Synaptophysin, VGLUT1, VGLUT2, VGAT, NR2B) were investigated, considering the known synaptogenic activity of Slitrk4 protein. As a result, immunopositive-particle-signal-intensity of PSD95 (−15%, *P* = 0.041) and VGAT (−17%, *P* = 0.031) decreased, while the others were comparable (Figures 7A–C). The particle signal density and size were comparable for all markers except that PSD95, the size of which slightly decreased (−1.1%, *P* = 0.031; Supplementary Figure 7). We next examined the markers for GABAergic interneuron subtypes, considering the electrophysiology results. The immunostaining-based cell counting revealed that a strong reduction of calretinin (CR)-expressing cell numbers in the anterior LA of *Slitrk4* KO mice (−77%, *P* = 8.1 × 10^−6^; in the entire LA, −33%, *P* = 0.040), while the numbers of neither calbindin (CB) nor parvalbumin-expressing cells were significantly different from those of WT (Figure 7D; Supplementary Figure 8). We also examined the selected transcript levels in the LA by collecting frozen punches (Figure 7E). The analysis revealed a significant reduction in some transcripts commonly produced in GABAergic interneurons, GAD65 (−49%, *P* = 0.019), GAD67 (−41%, *P* = 0.014), and VGAT (−42%, *P* = 0.042). Among the validated interneuron markers, a clear decrease was observed in the gastrin-releasing peptide receptor (GRPR) (−30%, *P* = 0.0096) (Figure 7E), which is known to be expressed in amygdala feedback-type interneurons (Shumyatsky et al., 2002).

Because the above marker analysis indicated a mild reduction in the excitatory postsynaptic marker, PSD95, we morphologically examined the spines of the amygdala principal neurons by Golgi-staining (Supplementary Figure 9). However, we did not observe differences in spine length, width, or density between WT and KO mice. We also tested the neurite morphology of hippocampal neurons, where a dense distribution of the Slitrk4 transcript was observed (Allen Brain Atlas, https://mouse.brain-map.org/experiment/show/74882938). The analysis of GFP-transfected cells did not show clear genotype-dependent differences (Supplementary Figure 10). We hypothesized that excitatory synapse- or neurite-associated roles were difficult to observe in *Slitrk4* KO mice, and focused on the presumptive role of Slitrk4 in GABAergic neuron subset development.

### 3.6 Impaired CR neuron development in Slitrk4 KO cerebral cortex

**Figure 8:**
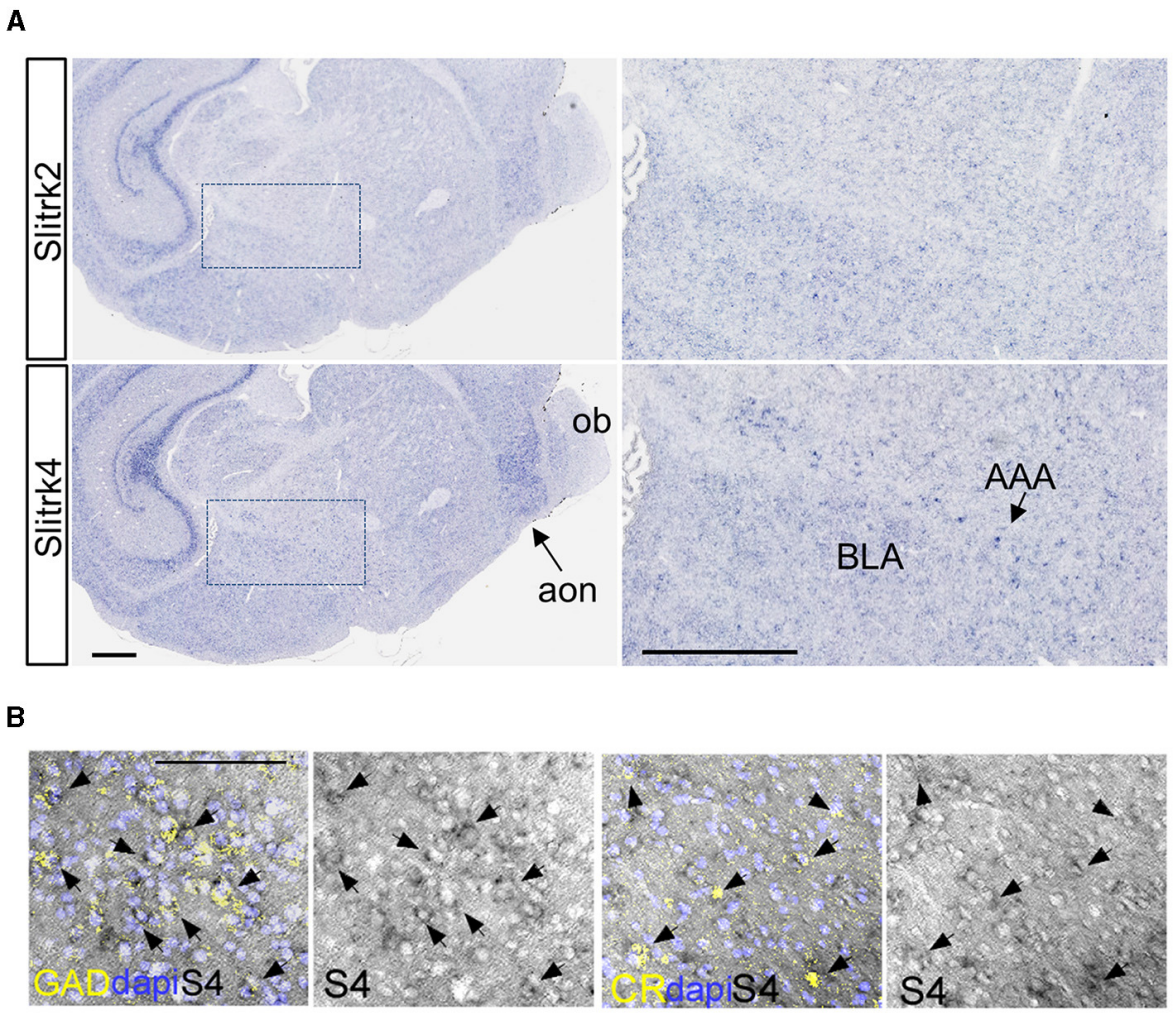
*Slitrk4* expression in early postnatal inhibitory interneurons. **(A)** Slitrk4 mRNA distribution in the amygdala and anterior olfactory nucleus in P12 mouse brain. The sections were prepared through a tilted sagittal plane. The right panels indicate the higher magnification of the *boxed region* in the left panels. Slitrk2 mRNA shows a similar expression profile to Slitrk4 mRNA, indicated as a reference (top panels). AAA, anterior amygdaloid area; BLA, basolateral amygdala; ob, olfactory bulb; aon, anterior olfactory nucleus. Scale bars, 500 μm. **(B)** Double labeling *in situ* hybridization for Slitrk4/GAD65 and Slitrk4/CR are carried out on coronal sections through P12 anterior amygdaloid area. *GAD*, GAD65 mRNA; *CR*, calretinin mRNA; *S4*, Slitrk4 mRNA. *Arrows* indicate Slitrk4^+^GAD65^+^ or Slitrk4^+^CR^+^ cells. Scale bar, 100 μm.

To further clarify the abnormalities of CR^+^ neurons in *Slitrk4* KO mice, we counted the CR^+^ cells in the amygdala at P0 but did not observe clear differences (Supplementary Figure 11). This result led us to the hypothesize that CR neuron-associated abnormalities become apparent during postnatal development. We first examined the distribution of Slitrk4 transcripts on postnatal days 1, 9, 12, and 14 mouse brains [Figures 8A, B (P12); Supplementary Figure 12 (P1, 9, 14)], and found that Slitrk4 mRNA-derived signals were enhanced in the amygdala, hippocampus, and anterior olfactory nucleus with moderate expression in the brain, including the cerebral cortex, on P9, 12, and 14. At P12 anterior amygdaloid area, we detected strongly Slitrk4-expressing cells that were partially overlapping with GAD65 or CR expressing cells (Figure 8B; 30% of Slitrk4^+^ cells were GAD65^+^; 13% of GAD65^+^ cells were Slitrk4^+^; 38% of Slitrk4^+^ cells were CR^+^; 43% of CR^+^ cells were Slitrk4^+^).

**Figure 9:**
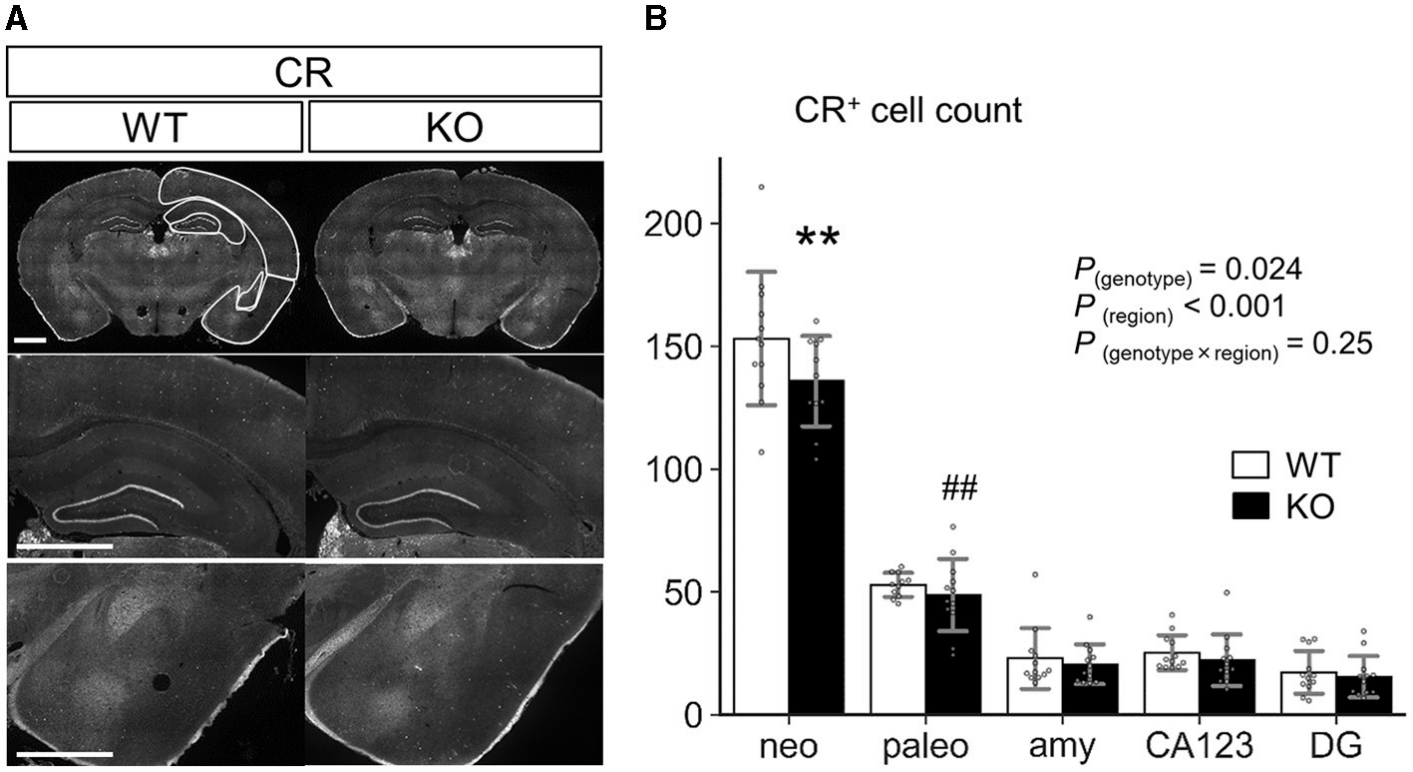
Quantification of CR^+^ cells in the cortex, amygdala, and hippocampus. **(A)** Representative images for immunostaining analysis. Scale bar, 1 mm. **(B)** Numbers of CR^+^ cells were counted at P35 in each region of coronal sections equivalent to AP −1.34 to −2.18 mm from Bregma. (*n* = 11 or 12 mice, 4–6 sections per mouse). Neo, neocortex; paleo, paleocortex; amy, amygdala; *CA123*, CA1-3 of the hippocampus; *DG*, dentate gyrus of the hippocampus. *P*-values indicate those in two-way ANOVA (genotype × region). **P < 0.01 in *post-hoc* Tukey test; ^*##*^*P* = 0.0018 in *F*-test. Error bar, SD.

We also examined the distribution of CR^+^ cells in neocortex, paleocortex, entire region of amygdala, CA1-3 regions and dentate gyrus of hippocampus in coronal sections from P35 WT and *Slitrk4* KO mice (Figure 9). The results revealed that mild reduction (−7 to −12%) of CR^+^ cells in all of the examined regions [*P*_*genotype*_ = 0.024, *F*_(1,108)_ = 5.3, in two-way ANOVA, genotype and regions as main factors, *n* = 11 or 12 mice for each genotype], and the reduction in neocortex was significant (*P* = 0.0027, in *post-hoc* Tukey test).

### 3.7 GABAergic neuron-generating competency was impaired in *Slitrk4* KO embryonic stem (ES) cells

**Figure 10:**
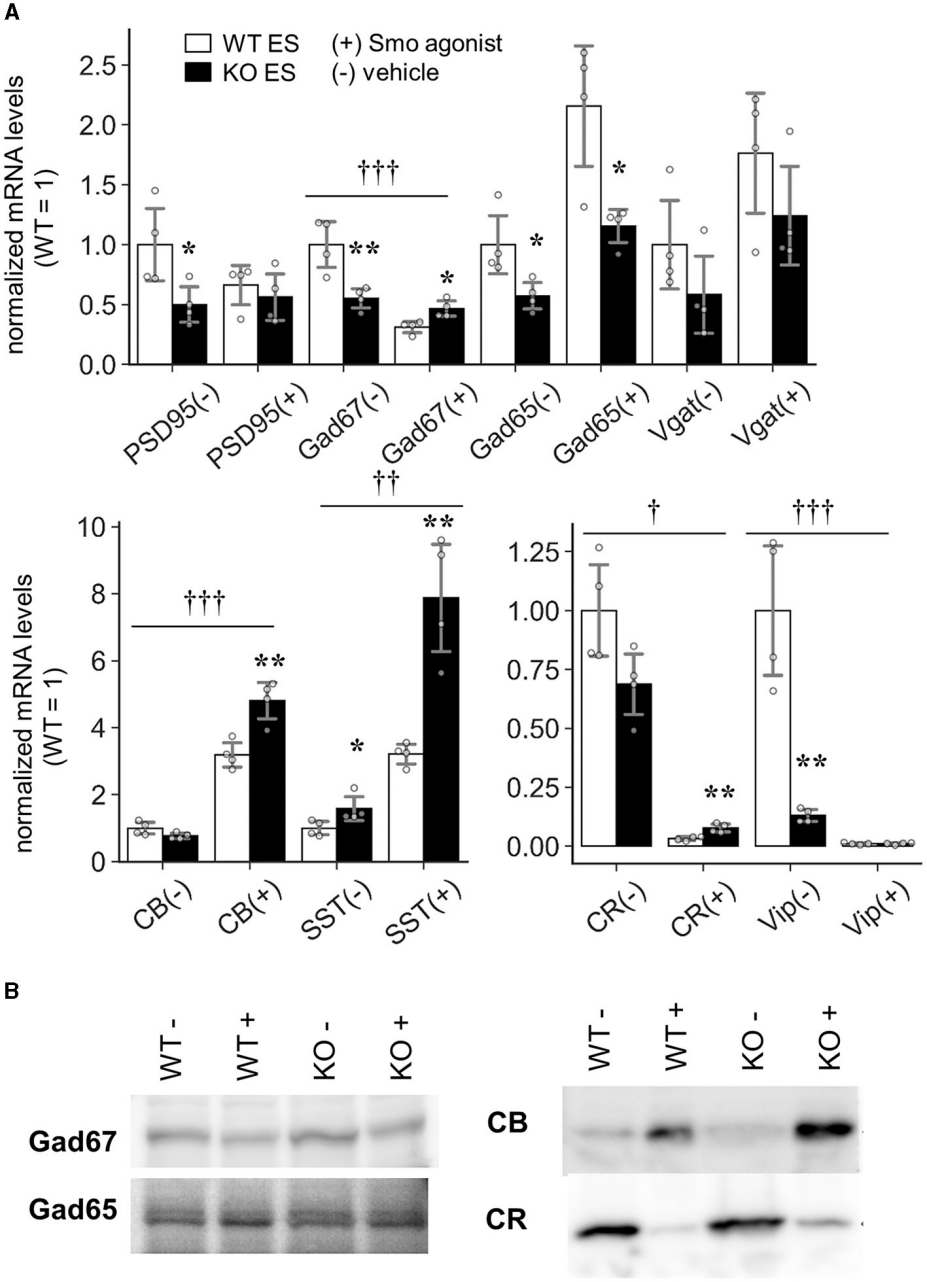
Induction of GABAergic interneurons in Slitrk4 KO ES cells. Slitrk4 WT or KO ES cells were subjected to *in vitro* neuronal differentiation with (+) or without (–) purmorphamine (Smo agonist). **(A)** Transcript levels were determined by quantitative PCR. Results are indicated as the means of four independent wells. Values were normalized to WT in each experiment (WT = 1). The analyzed genes are shown at the bottom of the graph. *P < 0.05; **P < 0.01 in Student's *t*-test between WT and KO ES cells. Error bar, SD.^†^*P* < 0.05; ^††^*P* < 0.01; ^†††^*P* < 0.001 in the two-way ANOVA (interaction of treatment and genotype). **(B)** Immunoblot images of the indicated proteins.

The above results revealed that the development of CR-positive GABAergic neurons was impaired in the *Slitrk4* KO brain. To prove that the abnormality is directly caused by *Slitrk4* deficiency, we tested whether *Slitrk4*-deficient ES can generate CR^+^ neurons (Figure 10). *Slitrk4* KO ES cells and their parental (WT) ES cells were differentiated into neurons with or without the sonic hedgehog signaling (Shh) activator purmorphamine (Smoothened agonist), which is known to affect GABAergic neuronal subtype determination (Tischfield and Anderson, 2017). In the *Slitrk4* KO cells induced neurons (iNs) without purmorphamine, PSD95, GAD67, and GAD65 transcripts were reduced among general excitatory or inhibitory markers (PSD95, −50%, *P* = 0.042; GAD67, −45%, *P* = 0.0095; GAD65, −43%, *P* = 0.032), and vasoactive intestinal peptide (VIP) was strongly reduced (−87%, *P* = 0.0016) and CR tended to be reduced (−31%, *P* = 0.059) among the GABAergic subset markers. In the presence of purmorphamine, both CB and somatostatin (SST) were strongly induced in WT iNs (CB, +218%, *P* = 7.8 × 10^−5^; SST, +221%, *P* = 3.5 × 10^−5^, compared to WT without purmorphamine), but further increased in *Slitrk4* KO iNs (CB, +51%, *P* = 0.0049; SST, +145%, *P* = 0.0026, compared to WT with purmorphamine). The increase in SST expression by purmorphamine agrees with the known fact that purmorphamine increases SST-expressing cells in iNs (Tischfield and Anderson, 2017). When we analyzed the genotype dependency of the purmorphamine effects on the marker gene expression, GAD67, CB, SST, CR, and VIP expression showed differential responses between WT and KO mice (*P* < 0.05, two-way ANOVA interaction of treatment and genotype) (Figure 10A). The results suggested that Slitrk4 has a functional linkage at some points downstream of the Shh signal directing GABAergic neuronal subtype specification.

## 4 Discussion

### 4.1 Behavioral abnormalities in Slitrk4 KO and their associated neural circuits

This study revealed several behavioral abnormalities in *Slitrk4* KO mice through a test battery consisting of 21 tests. The abnormalities are categorized into those related to social behavior, enhanced olfactory function, and enhanced fear memory, which would be helpful to consider the role of Slitrk4 in neural circuits together with Slitrk4 expression profile that is enhanced in the olfactory bulb, anterior olfactory nucleus, piriform cortex, amygdala, and periaqueductal gray matter at both protein and transcript levels (Figure 1; Supplementary Figure 2; Allen Brain Atlas).

Abnormalities in social behavior in *Slitrk4* KO mice are observed in two independent experimental paradigms that involve reciprocal interactions between freely moving mice (resident-intruder test in homecages and social interaction in a novel environment), but not in the approach to the caged mouse (social discrimination test). In addition, whereas hiding behavior was increased, the approaching behavior (contact) was not altered in the resident-intruder test. These results suggest that social memory or social investigation is not affected by loss of Slitrk4. We speculate that *Slitrk4* KO mice avoid social threats, reflecting presumptive abnormalities in social fear. Activation of the fear system leads to a series of behavioral and physiological responses that enable the animal to avoid a potentially dangerous situation (avoidance behaviors) (Toth and Neumann, 2013). Although rodents with social avoidance behavior are often accompanies with increased general anxiety and depression-like behaviors (Toth and Neumann, 2013), depression-like behavior may not be clear in *Slitrk4* KO mice. Because the increased immobility in both tail-suspension and forced swimming is limited to the first 1 min without any clear differences in the remaining test period.

The enhanced olfactory function is suggested solely by hidden cookie test. Additional verification may be needed for definite conclusion. However, the phenotype is intriguing in terms of Slitrk4 expression profile. Among the regions with strong Slitrk4 expression, anterior olfactory nucleus is known to modulate olfactory sensitivity and olfaction-dependent behavior by sending its inhibitory output to olfactory bulb granule neurons (Aqrabawi et al., 2016), which is important for social olfactory cue recognition (Oettl et al., 2016). Therefore, some functional alterations are predicted to be in the anterior olfactory nucleus or related neural circuits of *Slitrk4* KO mice.

As to the enhanced auditory cue-associated fear memory acquisition, the role of amygdala has been well-recognized (LeDoux, 2000; Ehrlich et al., 2009; Johansen et al., 2011; Duvarci and Pare, 2014). Besides the abnormalities in lateral nucleus, which we discuss below, the other regions with strong Slitrk4 may is involved in the behavioral abnormalities in *Slitrk4* KO mice. In particular, the piriform cortex is a site of olfactory information processing (Stettler and Axel, 2009) and is essential for odor fear memories (Meissner-Bernard et al., 2019), and periaqueductal gray matter is associated with defensive responses (Motta et al., 2017).

It should be noted that the three behavioral abnormalities in *Slitrk4* KO mice are closely related. The enhanced olfactory function may be associated with social behavior abnormalities in *Slitrk4* KO mice because olfactory cues are essential for rodent social behaviors (Crawley, 2007; Ryan et al., 2008; Bourne et al., 2013), and enhanced fear memory and fear conditioning by foot shock impairs rodent social behaviors (Toth and Neumann, 2013). Furthermore, the amygdala neural circuit is known to modulate social behavior (Janak and Tye, 2015). Thus, we consider that the behavioral phenotypes of *Slitrk4* KO mice could be summarized briefly as the enhanced olfactory and fear processing systems.

### 4.2 Altered synaptic function in the amygdala neural circuit of *Slitrk4* KO mice

We focused on the amygdala neural circuit to elucidate the role of Slitrk4 because amygdala neural circuit properties have been well-correlated with various aspects of fear memory owing to many excellent studies (LeDoux, 2000; Janak and Tye, 2015). Our results indicated that increased LTP with reduced feedback inhibition underlies enhanced fear memory in *Slitrk4* KO mice. Accordingly, recent studies have revealed the essential roles of inhibitory neurons in amygdala neural circuits (Krabbe et al., 2018). Interneuron subtypes involved in either feedback or feedforward-type inhibition have been investigated in several studies (Shumyatsky et al., 2002; Bissiere et al., 2003; Samson et al., 2003; Lee et al., 2013; Wolff et al., 2014; Unal et al., 2020). Grpr^+^ neurons (Shumyatsky et al., 2002) and SST^+^ neurons (Unal et al., 2020) have been proposed to be responsible for feedback inhibition. The latter study fluorescently labeled living SST^+^ interneurons and reported that SST^+^ interneurons with low threshold spiking mediate feedback inhibition.

Cortical CR^+^ interneurons constitute 10–30% of all cortical neurons and can be divided into two main populations: CR^+^VIP^+^ neurons derived from the caudal ganglionic eminence (CGE) and CR^+^SST^+^ neurons from the dorsal part of the medial ganglionic eminence (dMGE) (Cauli et al., 2014; Hu et al., 2017). The development of dMGE-derived interneurons requires proper Shh signaling (Flandin et al., 2011). Because we observed abnormalities of CR^+^ neurons in the neocortex and amygdala of *Slitrk4* KO mice, we consider that CGE- or dMGE-derived CR^+^ neurons are affected in *Slitrk4* KO mice. Considering Shh signaling-induced changes in interneuron marker expression were affected by *Slitrk4* deficiency (Figure 10), abnormalities of dMGE-derived CR^+^ neurons are presumed. In previous studies, MGE-restricted ablation of Shh was shown to increase apoptosis of MGE on embryonic day 18 and reduce both the numbers of CR^+^ cells and SST^+^ cells in P24 cortex (Flandin et al., 2011), and Smo inactivation of MGE converts the cell fate conversion to CGE-derived CR^+^VIP^+^ like cells (Xu et al., 2010). The timing of CR^+^ neuron decrement in *Slitrk4* KO mice (between P0 and P35) seems to fit with the hypothetical abnormalities in dMGE-derived CR^+^ neurons.

However, the involvement of CGE-derived CR^+^ neurons cannot be ruled out at this point. In recent RNA-seq-based databases (Saunders et al., 2018; Zeisel et al., 2018; Hochgerner et al., 2023), CR^+^VIP^+^ interneurons highly express Grpr and Ptprd (e.g., Interneuron_CGE_Cplx3-Synpr[#4] in the DropViz database) (Mayer et al., 2018). On the other hand, MGE-derived amygdala SST^+^ neurons contain both CR^+^ subgroup and CR^+^Grpr^+^ subgroups (Hochgerner et al., 2023). Because Grpr expression in the amygdala was reduced in the LA of *Slitrk4* KO mice, a further detailed analysis may be needed for conclusion.

Concerning the mechanism underlying Slitrk4-mediated control of CR^+^ neuron development, the interaction with Shh signaling or Ptprd signaling seems potent at present. Shh signaling organizes ventral neural tissue development at various sites and stages with key regulators, such as protein kinase A (Bertrand and Dahmane, 2006; Álvarez-Buylla and Ihrie, 2014; Yabut and Pleasure, 2018). Meanwhile, Ptprd is known as a tumor suppressor that is inactivated and mutated in glioblastoma and other human cancers (Veeriah et al., 2009) in addition to its role in presynapse maturation, controlling STAT3 signaling (Uhl and Martinez, 2019) which is involved in a broad range of cell differentiation (Hirano et al., 2000; Kortylewski et al., 2005; Li et al., 2020; Tomita et al., 2020). Further investigation is expected to link the Slitrk4 molecular function to candidate signaling pathways.

In terms of known molecular function, Slitrk4 possesses neurite-modulating activities (Aruga and Mikoshiba, 2003; Marteyn et al., 2011) and synaptogenic activities (Takahashi et al., 2012; Yim et al., 2013). Mouse Ptprd can bind the Slitrk4 extracellular domain or LRR1 domain in a pull-down assay (Yamagata et al., 2015), and human SLITRK4 has been identified as a binding partner for Ptprd by surface plasmon resonance analysis (Verschueren et al., 2020). Ptprd is a membrane-spanning-type protein tyrosine phosphatase that is essential for the integrity of presynapse (Takahashi and Craig, 2013). As synapse-related abnormalities, we observed reduced PSD95-immunopositive signals in the LA of *Slitrk4* KO mice (Figure 7; Supplementary Figure 7) without any abnormalities in spine shapes of the LA neurons (Supplementary Figure 9). The neurite shapes of dissociated hippocampal neurons is not clearly affected in *Slitrk4* KO mice (Supplementary Figure 10). As a whole, the evidence may be limited in current results to consider the neurite-modulating or synaptogenic activities of *Slitrk4* gene *in vivo*.

### 4.3 Clinical implications of the current results

The results of this study may have important clinical implications. First, Slitrk4 has been associated with neuropsychiatric disorders, as was the case for the other Slitrk family genes (Proenca et al., 2011); the results of this study show that Slitrk4 is a critical gene that suppresses fear-associated behaviors, raising the possibility of its involvement in PTSD or phobia-associated diseases. Supporting this idea, a significant upregulation of SLITRK4 was found in the female prefrontal cortices (orbitofrontal cortex and subgenual prefrontal cortex) of patients with PTSD (Girgenti et al., 2021) and the blood of military personnel with PTSD (Guardado et al., 2016). Second, function-damaging mutations have been identified in SLITRK4 in patients with schizophrenia patients (Piton et al., 2011; Kang et al., 2016). Abnormalities of GABAergic interneurons have been described in postmortem schizophrenia patients. Broad expression of Slitrk4, including the cerebral cortex, and mildly decreased prepulse inhibition in *Slitrk4* KO mice may be in line with its involvement in schizophrenia pathophysiology. Third, we did not observe DM1-related behavioral abnormalities such as hypotonia and hypersomnolence (Gomes-Pereira et al., 2011) in *Slitrk4* KO mice. However, some central nervous system-related signs of DM1 such as cognitive impairment and executive dysfunction are not conclusive at this point. Fourth, Slitrk4 deregulation may be associated with oncogenesis. This is primarily because SLITRK4 physically interacts with the tumor suppressor Ptprd. However, this is also supported by the overexpression of SLITK4 in leiomyosarcoma (Davidson et al., 2014) and the identification of SLITRK4 as a causative gene in chronic myelocytic leukemia (Yamazaki et al., 2016) and hepatocellular carcinoma (Wu et al., 2020).

### 4.4 Limitations and perspectives

Although we focused the role of Slitrk4 in the amygdala neural circuit in this study, Slitrk4 may have critical roles in other neural circuits. In particular, those in the anterior olfactory nucleus attracts our attention. Because current studies showed the overall behavioral phenotypes of pan *Slitrk4* KO mice, further analysis adopting spatiotemporally restricted gene function modification would be necessary to obtain more clear views on the roles of *Slitrk4* in each neural circuit. In addition, such analyses should address the sex-effect and the gene dosage effect on phenotypes, analyses using female mice are absolutely needed because *Slitrk4* is located on X chromosome both in mice and humans (Aruga et al., 2003) and there is a sex bias in the PTSD transcriptome analysis (Girgenti et al., 2021).

Another unsolved problem is the actual molecular function of Slitrk4 underlying the Slitrk4 KO amygdala phenotype. Colocalization of excitatory PSD95 and reduction of PSD95 punctate signals in *Slitrk4* KO amygdala suggest that Slitrk4 could have a role in maintaining synaptic integrity in amygdala neural circuit. Although we have shown the decrement of GABAergic neuron subset in this study, this phenotype could be explained by loss of presumptive synaptogenic function of Slitrk4 or by other unknown functions of Slitrk4. For the former, the reduction of PSD95 punctate signals implies alterations in AMPA receptor and NMDA receptor components. Investigating these possible alterations could significantly contribute to a more comprehensive understanding. For the latter, it is interesting that another member of Slitrk family, Slitrk5 is present in primary cilium and acts as negative regulator of hedgehog signaling in osteoblasts (Sun et al., 2021).

As a clue for above challenges, it would be essential to clarify the localization of Slitrk4 protein. Although we generated antibody against Slitrk4 in this study, there remained technical limitation to detect endogenous Slitrk4 protein in immunostaining.

Finally, we provided a model in which a GABAergic interneuron subset was downregulated in this study. Although recent studies have focused on CR-expressing subgroups, the physiological roles of other GABAergic subsets remain to be clarified. Based on the mature universal classification, which unifies several RNA-seq studies of neural cells, a better understanding is expected in the near future. In terms of gene function investigation, the current results shed new light on the role of Slitrk family proteins in the regulation of cell-type specification or maintenance. Considering the tissue expression profiles of Slitrk family genes, including hematopoietic stem cells and leukemia (Milde et al., 2007) and brain tumors (Aruga et al., 2003), its role in development and oncogenesis awaits further investigation.

## Data availability statement

The original contributions presented in the study are included in the article/Supplementary material, further inquiries can be directed to the corresponding author.

## Ethics statement

The animal study was approved by Animal Experiment Committees at the RIKEN Brain Science Institute and Animal Care and Use Committee of Nagasaki University. The study was conducted in accordance with the local legislation and institutional requirements.

## Author contributions

YM: Conceptualization, Investigation, Writing – original draft, Writing – review & editing. HM: Conceptualization, Investigation, Writing – original draft, Writing – review & editing. K-iK: Conceptualization, Investigation, Writing – original draft, Writing – review & editing. AW: Investigation, Writing – original draft, Writing – review & editing. KY: Investigation, Writing – original draft, Writing – review & editing. TI: Investigation, Methodology, Writing – original draft, Writing – review & editing. SN: Conceptualization, Investigation, Writing – original draft, Writing – review & editing. JA: Conceptualization, Funding acquisition, Investigation, Supervision, Validation, Writing – original draft, Writing – review & editing.
