# Supplementary material for: Slitrk4 is required for the development of inhibitory neurons in the fear memory circuit of the lateral amygdala
Source: Front Mol Neurosci. 2024 Apr 26;17:1386924. doi: 10.3389/fnmol.2024.1386924 (PMC11082273; doi:10.3389/fnmol.2024.1386924)
Supplement: Supplementary file 1 [file Data_Sheet_1.PDF]

Primers for RT-PCR analysis

Ptprd_F	TGACAAACTGCAGGATGGTG
Ptprd_R	CCGGAGTTCGTGTAAACCTG
Gad65_F	TGTAGCTGACATCTGCAAAAAGTA
Gad65_R	GGGACATCAGTAACCCTCCA
Gad67_F	ATACAACCTTTGGCTGCATGT
Gad67_R	TTCCGGGACATGAGCAGT
Vgat_F	ACGTGACAAATGCCATTCAG
Vgat_R	TGAGGAACAACCCCAGGTAG
Vglut1_F	GTGCAATGACCAAGCACAAG
Vglut1_R	AGATGACACCGCCGTAGTG
Vglut2_F	GGAAAATCCCTCGGACAGA
Vglut2_R	TGGTCTCTCGGTTGTCCTG
PSD95_F	TCTGTGCGAGAGGTAGCAGA
PSD95_R	CGGATGAAGATGGCGATAG
NR2B_F	GGGTTACAACCGGTGCCTA
NR2B_R	CTTTGCCGATGGTGAAAGAT
Gabra2_F	ACAAAAAGAGGATGGGCTTG
Gabra2_R	TCATGACGGAGCCTTTCTCT
Gabra3_F	CTTGGGAAGGCAAGAAGGTA
Gabra3_R	TGGAGCTGCTGGTGTTTTCT
Gabra5_F	GACGGACTCTTGGATGGCTA
Gabra5_R	ACCTGCGTGATTCGCTCT
Grpr_F	TGATTCAGAGTGCCTACAATCTTC
Grpr_R	CTTCCGGGATTCGATCTG
Calbindin_F	AAGGCTTTTGAGTTATATGATCAGG
Calbindin_R	TTCTTCTCACACAGATCTTTCAGC
Somatostatin_F	CCCAGACTCCGTCAGTTTCT
Somatostatin_R	GGGCATCATTCTCTGTCTGG
Calretinin_F	CGAAGAGAATTTCCTTTTGTGC
Calretinin_R	TGTGTCATACTTCCGCCAAG
VIP_F	GCCTCTCTTTGGACCACCTT
VIP_R	CTCCTTCAAACGGCATCCT

Supplementary Table 1. List of PCR primers

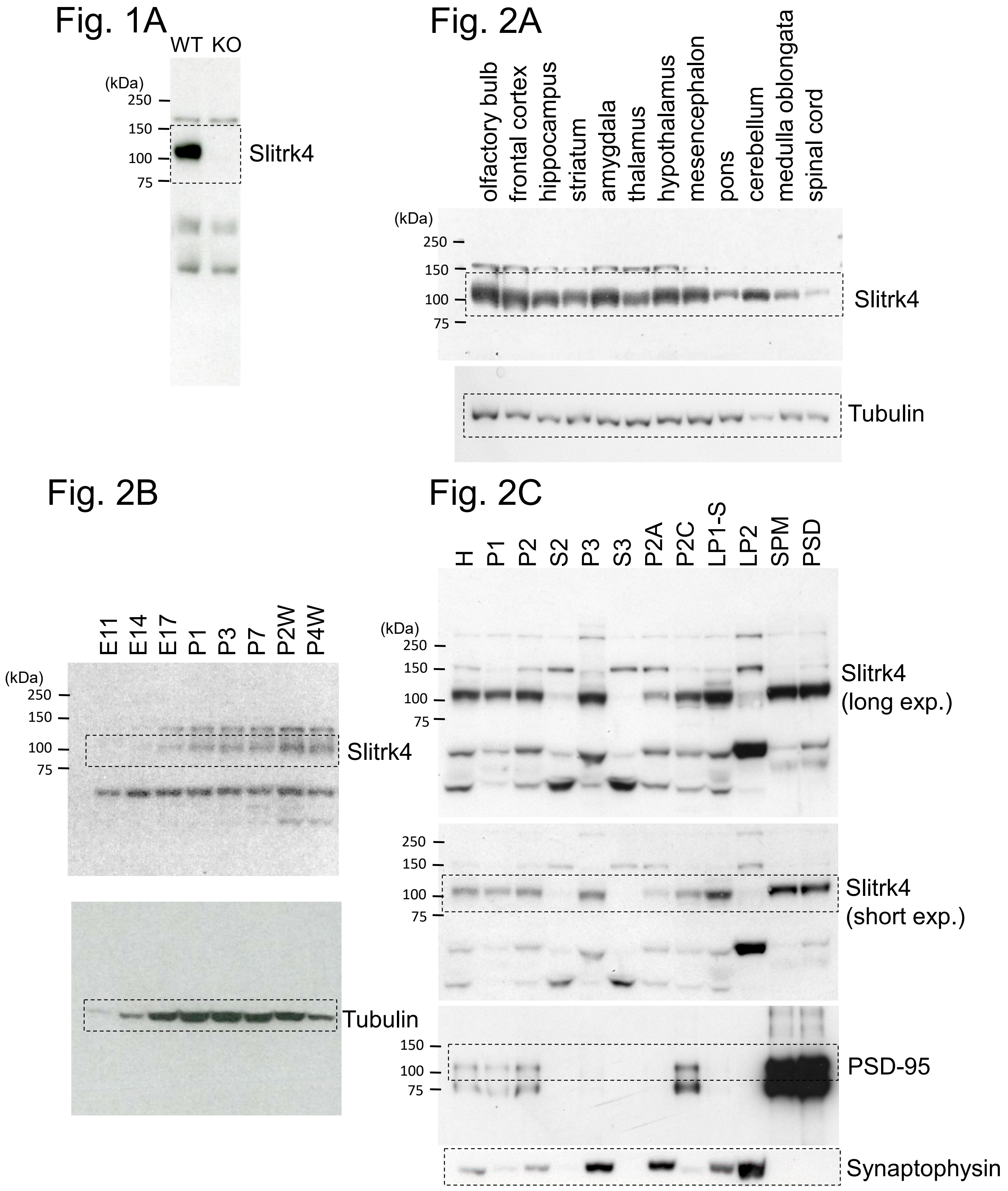

Supplementary Figure 1. Full immunoblot images for Fig. 1 and Fig. 2.

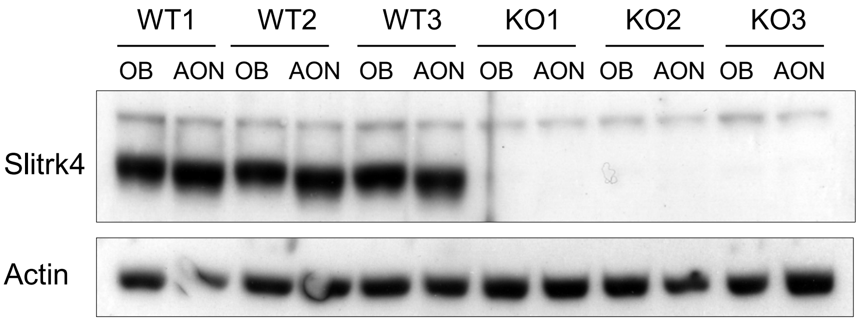

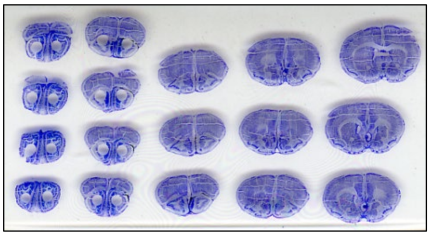

Supplementary Figure 2. Distribution of Slitrk4 in olfactory bulb and anterior olfactory nuclei.
Top, immunoblot using anti-Slitrk4 or anti-Actin antibody. *Bottom*, Cresyl violet staining of sections after sampling. The tissue was collected from the frozen sections (150 µm thickness) from 8 M-old male mice (WT, $n = 3$; KO, $n = 3$) using biopsy punch (1 mm).

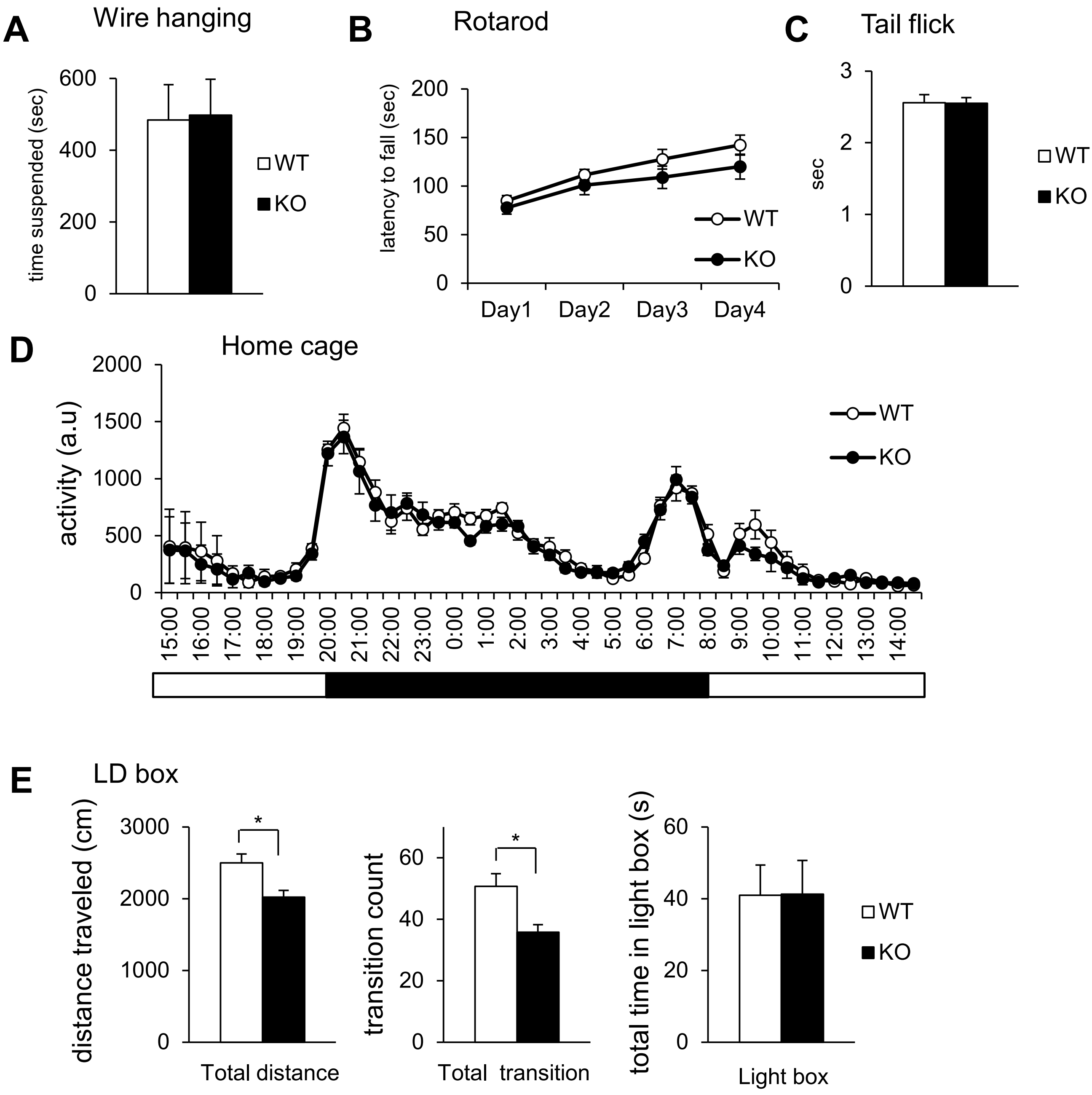

Supplementary Figure 3. Motor and sensory functions were not impaired in Slitrk4 KO mice. (A) Wire hanging test. Hanging wire times did not differ between WT and KO mice. (WT, *n*=10; KO, *n*=10). (B) Rotarod test. Presented is the latency to fall on the accelerating rod. Although there was no significant difference between WT and KO mice. (C) Tail flick test. (D) Homecage activity. Closed bar indicates dark phase. (E) Light-dark box test. Total distance (*Left*) and the total number of transition between light and dark box (*middle*) were significantly decreased in Slitrk4 KO mice. Total stay time in light box (*right*) was not different. *P < 0.05, **P < 0.01, Student t-test. Error bar, SEM. WT, *n* = 10; KO, *n* = 10.

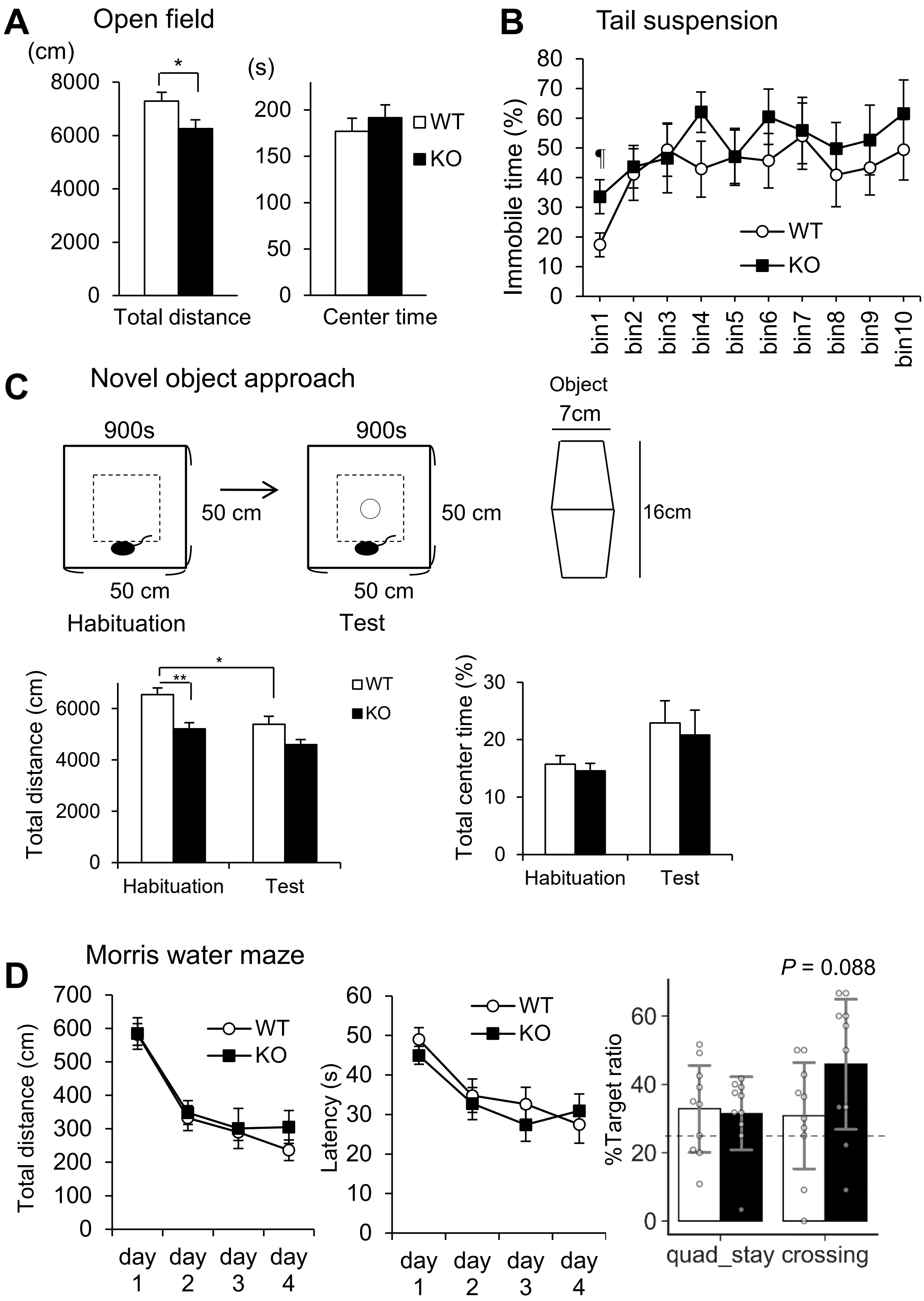

Supplementary Figure 4. Open field test, novel object approach test, and water maze test. (A) Open field test. Left: The total moved distance was reduced in Slitrk4 KO mice during test session (15 min). Right: Time spent in center area of open field was not different between WT and Slitrk4 KO mice. (B) Tail suspension test. Bin = 30 s. (C) Novel object approach test. (D) Morris water maze test. *Left*, total distance to reach platform; *middle*, latency to reach platform; *right*, Percentages of the target quadrant stayed time (*quad_stay*) and crossing over the target. Broken line, chance level. Error bar, SEM. WT, $n = 10$; KO, $n = 10$; $*P < 0.05$, $**P < 0.01$ in Student's t-test (A, C). ¶$P = 0.037$ in U-test, but not significant in post hoc Sidak test after rmANOVA (B).

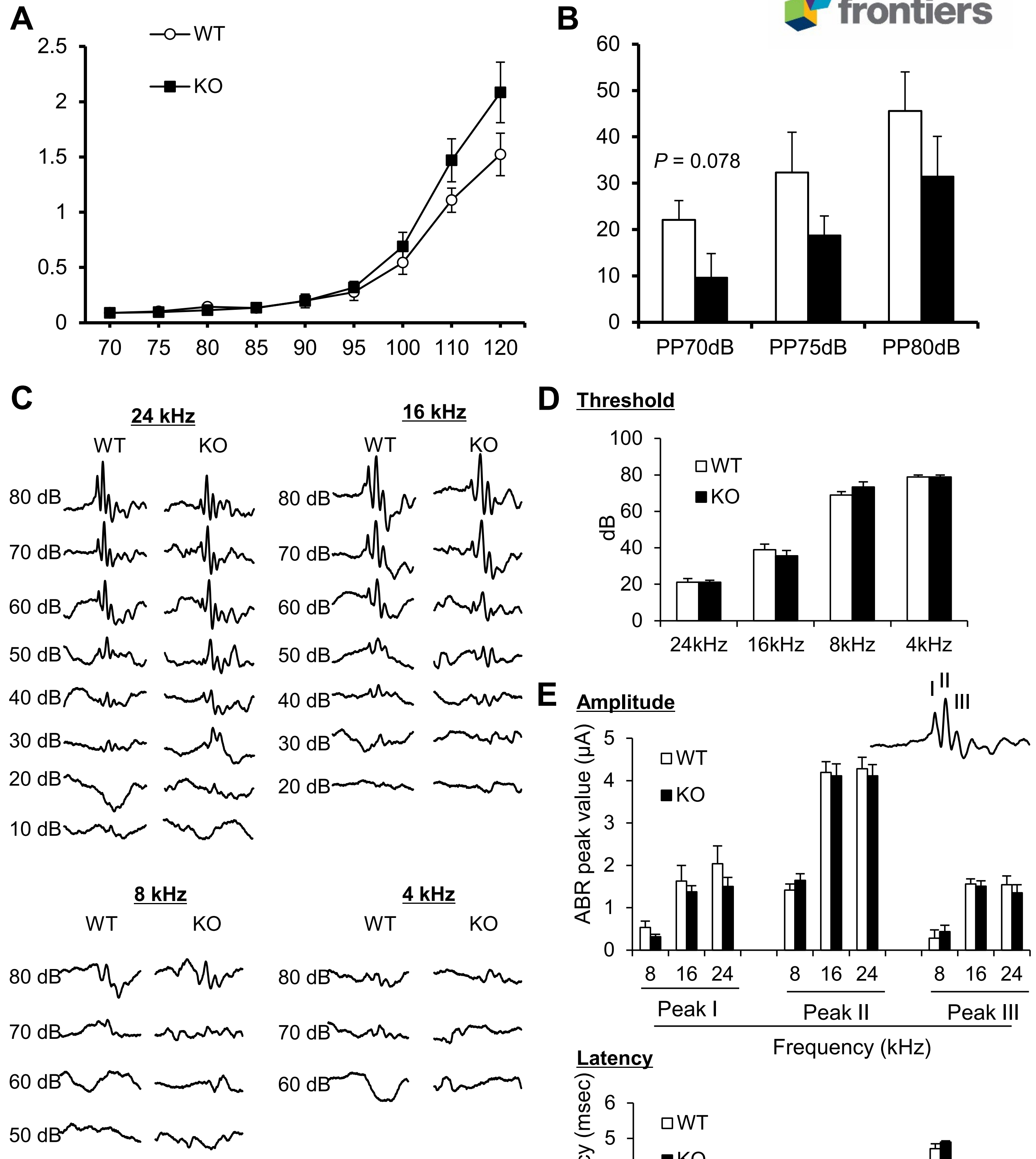

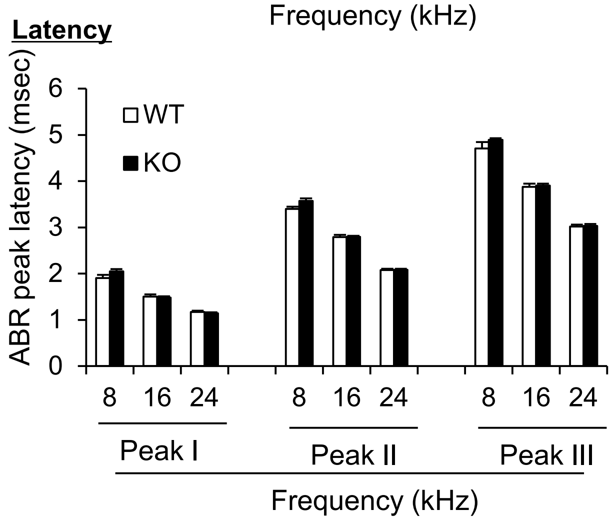

Supplementary Figure 5. Auditory function-associated tests in Slitrk4 KO mice.
(A) Audtitory startle response. (B) Prepulse inhibition of auditory startle response. (C) Representative waves of auditory brainstem response (ABR) from 8 weeks-old male WT (*left*, $n = 9$) and Slitrk4 KO (*right*, $n = 9$) mice. ABR were recorded upon 0- to 80-dB sound pressure level stimuli of 2, 4, 8, 16, and 24 kHz. The scale bars are shown at bottom right. (D) ABR thresholds in WT and Slitrk4 KO mice. (E) Each peak number is indicated in a representative ABR wave. *Top*, Comparison of the values of peaks I, II, and III between WT and Slitrk4 KO mice. *Bottom*: The latency to peaks I, II, and III. Error bar, SEM. *P* value was obtained in Student's *t*-test.

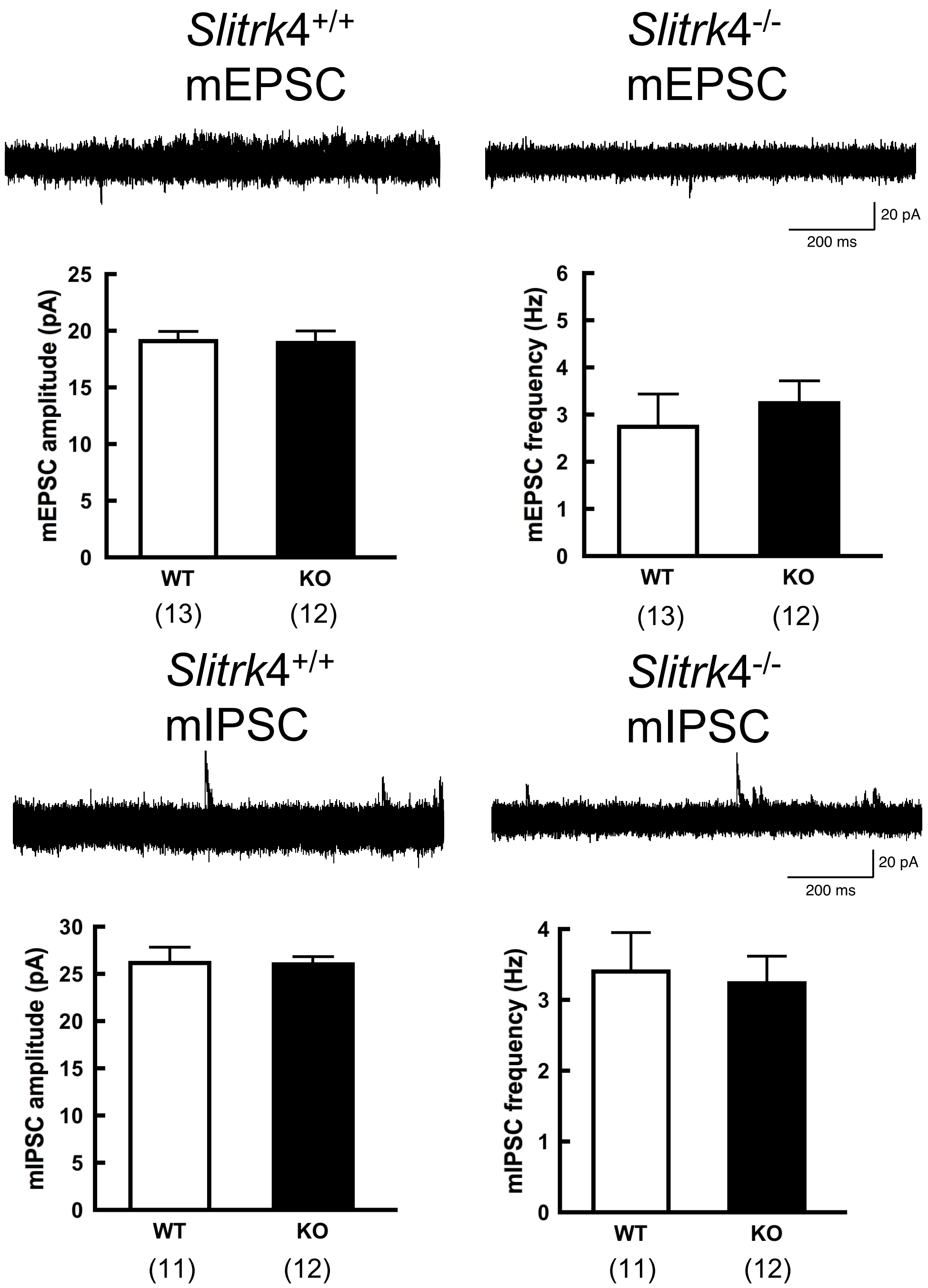

Supplementary Figure 6. Minimal postsynaptic currents. (*Top*) mEPSC. (*Bottom*) mIPSC. There was no difference in EPSP/IPSP ratio between WT and Slitrk4 KO mice. Numbers in parentheses indicate the number of slices. Error bar, SEM.

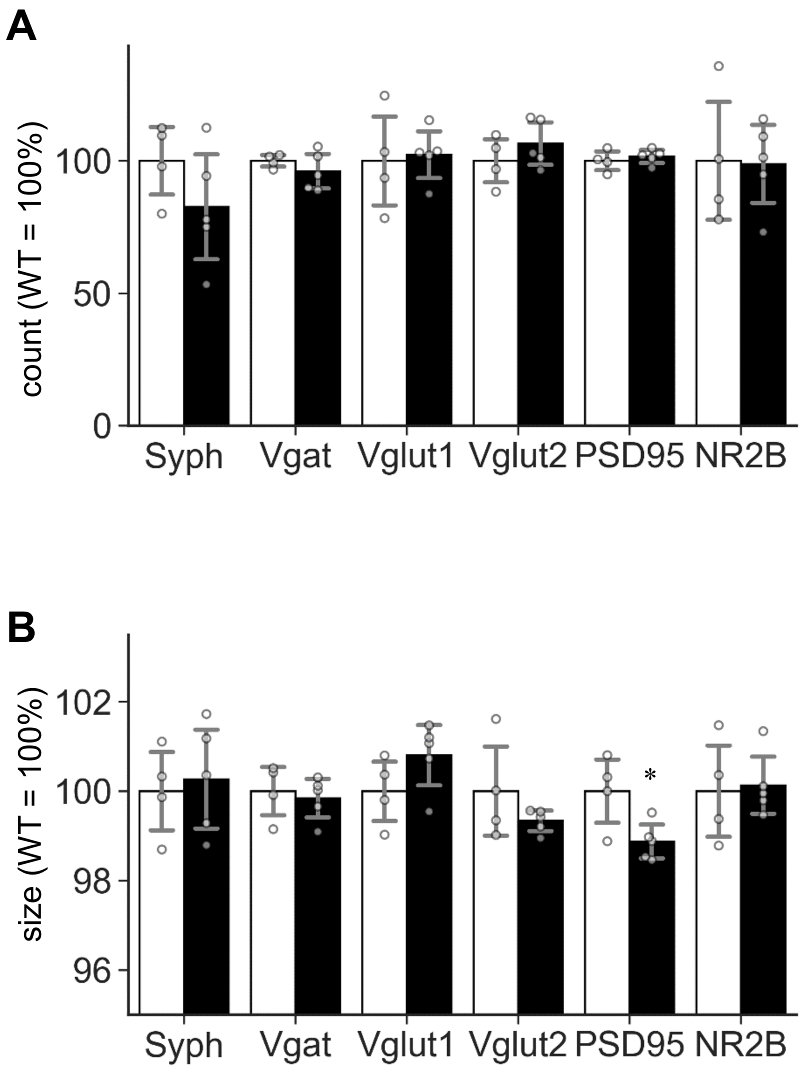

Supplementary Figure 7. Counts and sizes of immunopositive-particle-signals in LA. (A) Particle counts. (B) Particle size. WT, $n = 4$ mice, KO, $n = 5$ mice. Value for a mouse is the mean of eight images. Error bar, SD.*$P < 0.05$ in Student's *t*-test.

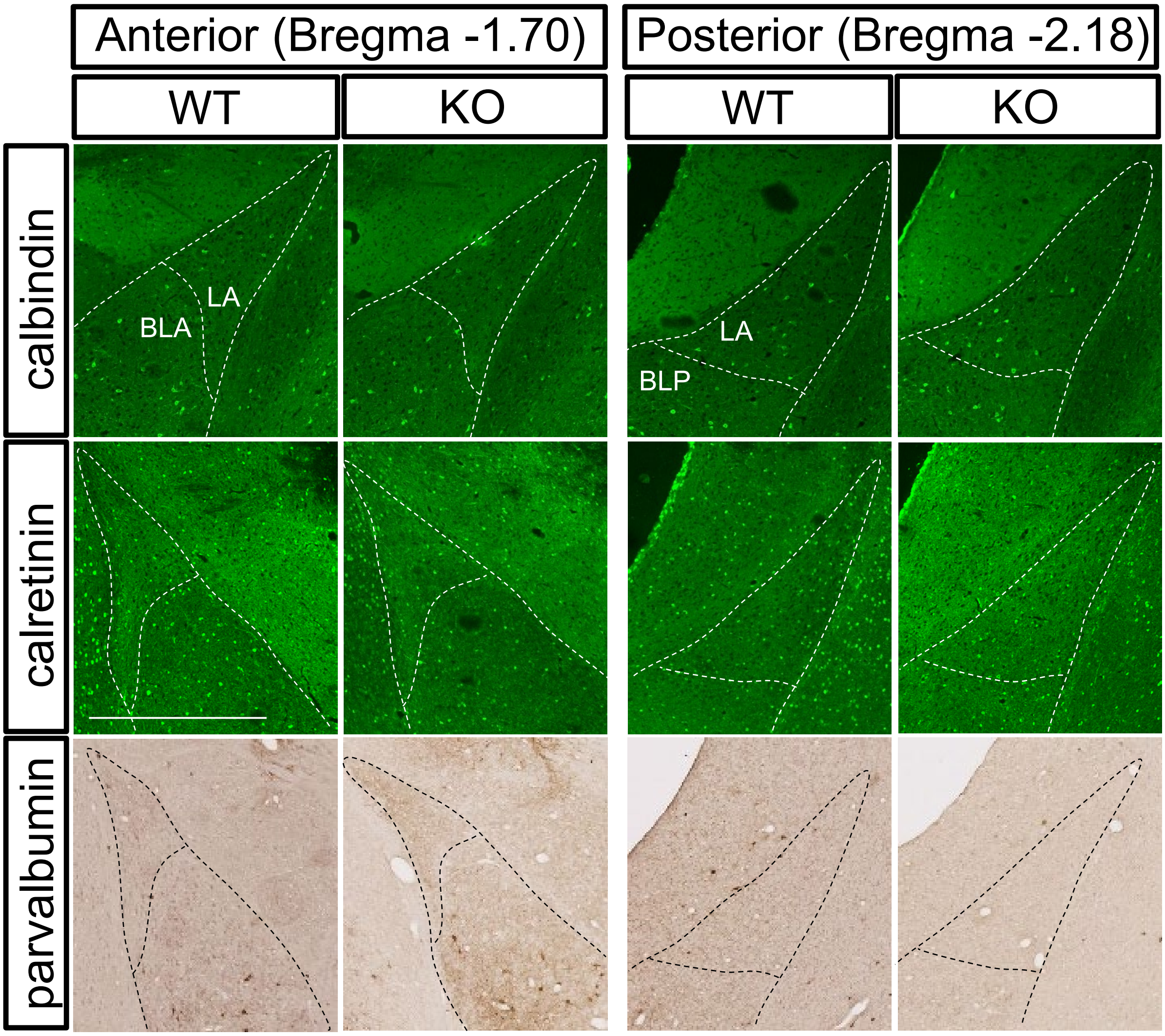

Supplementary Figure 8. Distribution of interneurons in Slitrk4 KO amygdalar lateral nuclei (LA). BLA, anterior basolateral nuclei; BLP, posterior basolateral amygadala nuclei. Reginal boundaries are based on Paxinos and Franklin (2001). Scale bar, 500 μm.

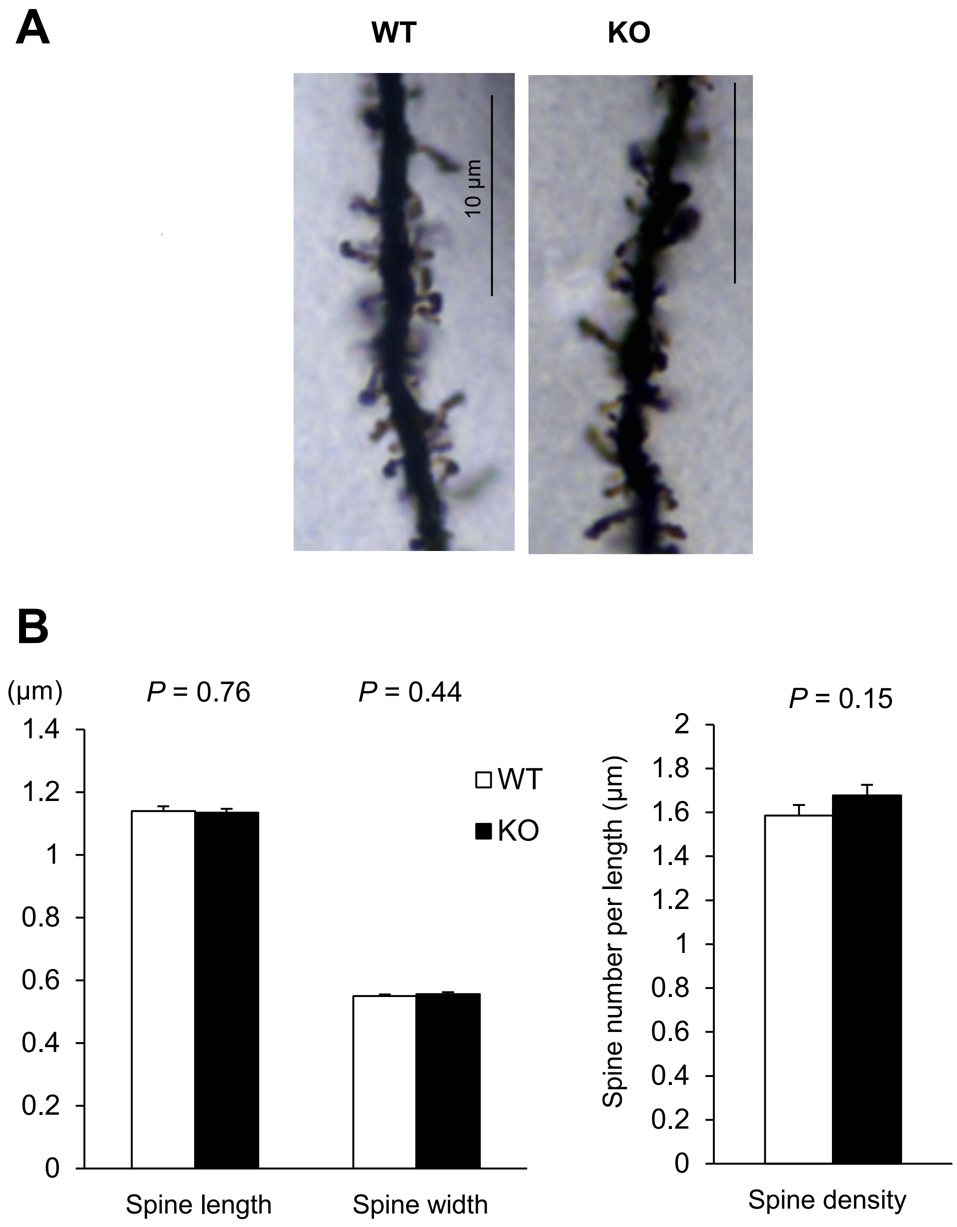

Supplementary Figure 9. Spine morphology was intact in Slitrk4 KO mice.
Golgi staining was carried out for 8 weeks-old brain coronal sections of 100 µm thickness. (A) Representative pictures of LA principal neurons proximal dendrites. (B) Mean spine length, width, and density. Error bar, SEM. WT, n = 97 branches from 3 mice including total 3484 spines; KO, n = 96 branches from 3 mice including total 3275 spines. None of the three parameters showed significant differences between WT and Slitrk4 KO mice.

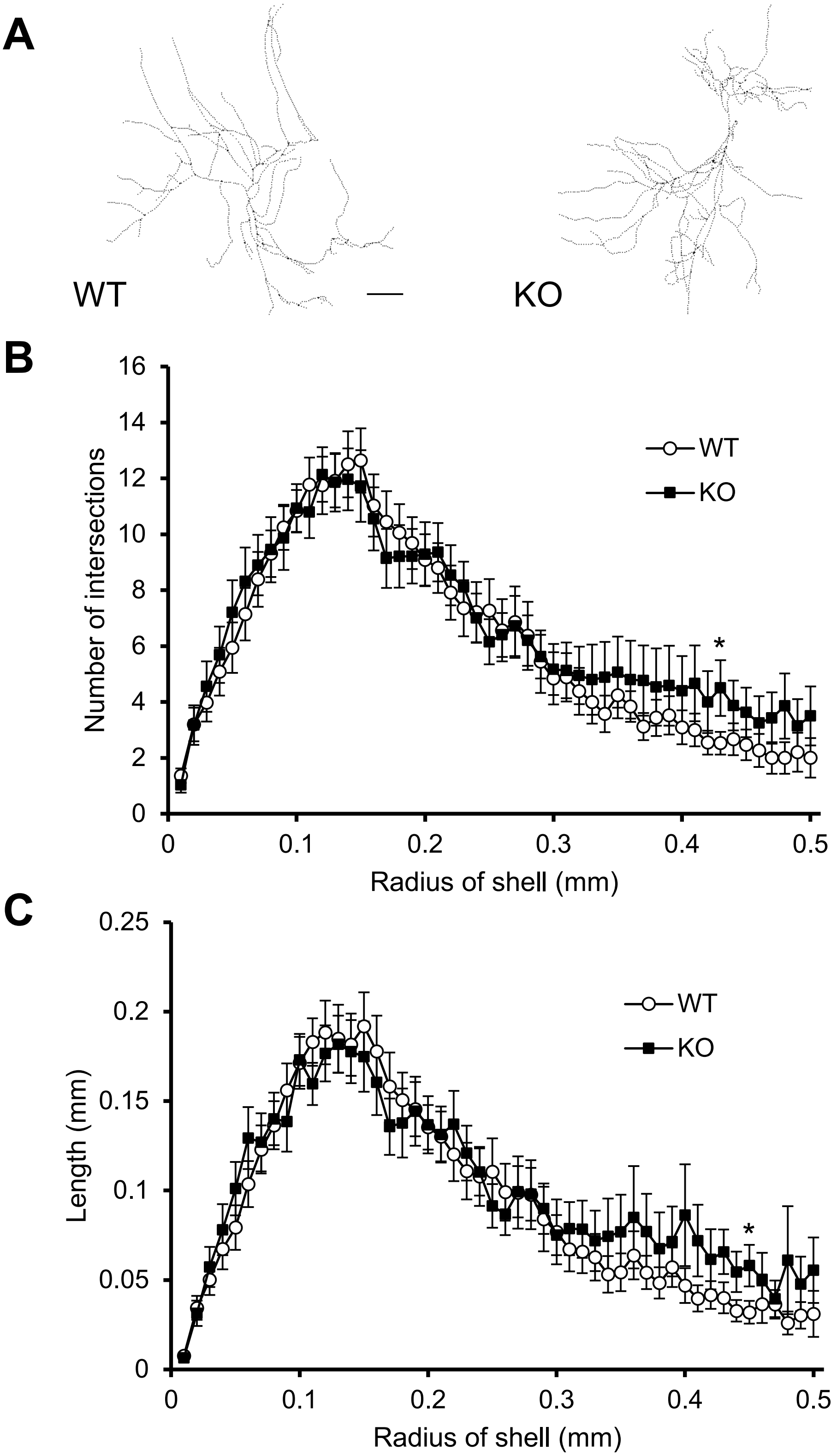

Supplementary Figure 10. Neurite morphology was comparable between WT and Slitrk4 KO-derived hippocampal neurons. The dissociated neurons in culture were transfected with pCAG-ires-GFP at DIV8, fixed at DIV 21, and immunostained with anti-GFP antibody. (A) Representative images of neurite tracing. (B, C) Sholl analysis of the traced images. (B) Number of intersections. (C) Neurite length. WT, $n = 36$ neurons from 7 mice; KO, $n = 29$ neurons from 6 mice. Error bar, SEM. Scale bar, 50 μm. $*P < 0.05$ in t-test.

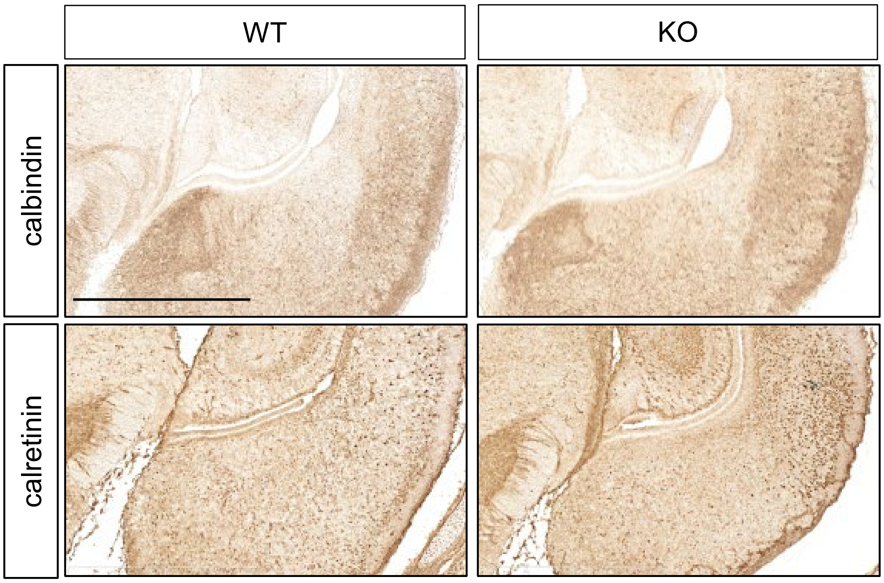

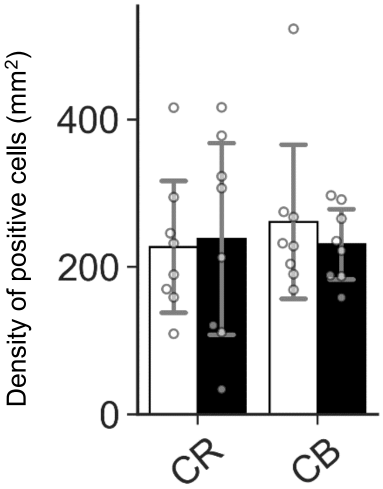

Supplementary Figure 11. Interneuron numbers were not altered in amygdala at newborn stage.
Immunostaining of Calretinin (CR) and Calbindin (CB) was carried out for newborn (P0) WT and Slitrk4 KO mice. *n* = 8 mice for each genotype. Error bar, SD. Scale bar, 1 mm.

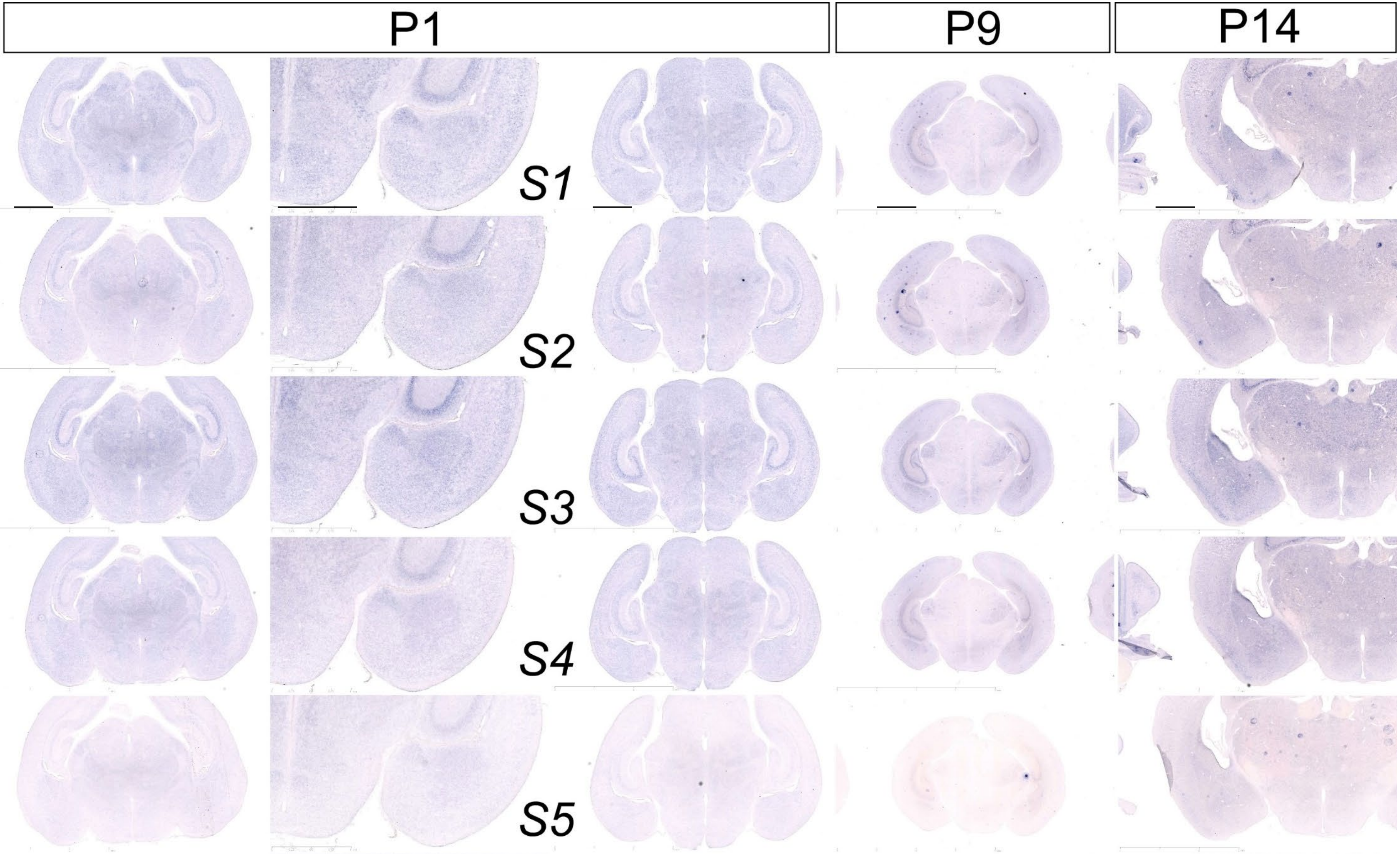

Supplementary Figure 12. Distribution of Slitrk1-Slitrk5 (S1-S5) mRNAs in developing mouse brain. Slitrk6 is not expressed in amygdala. Scale bars, 1 mm.

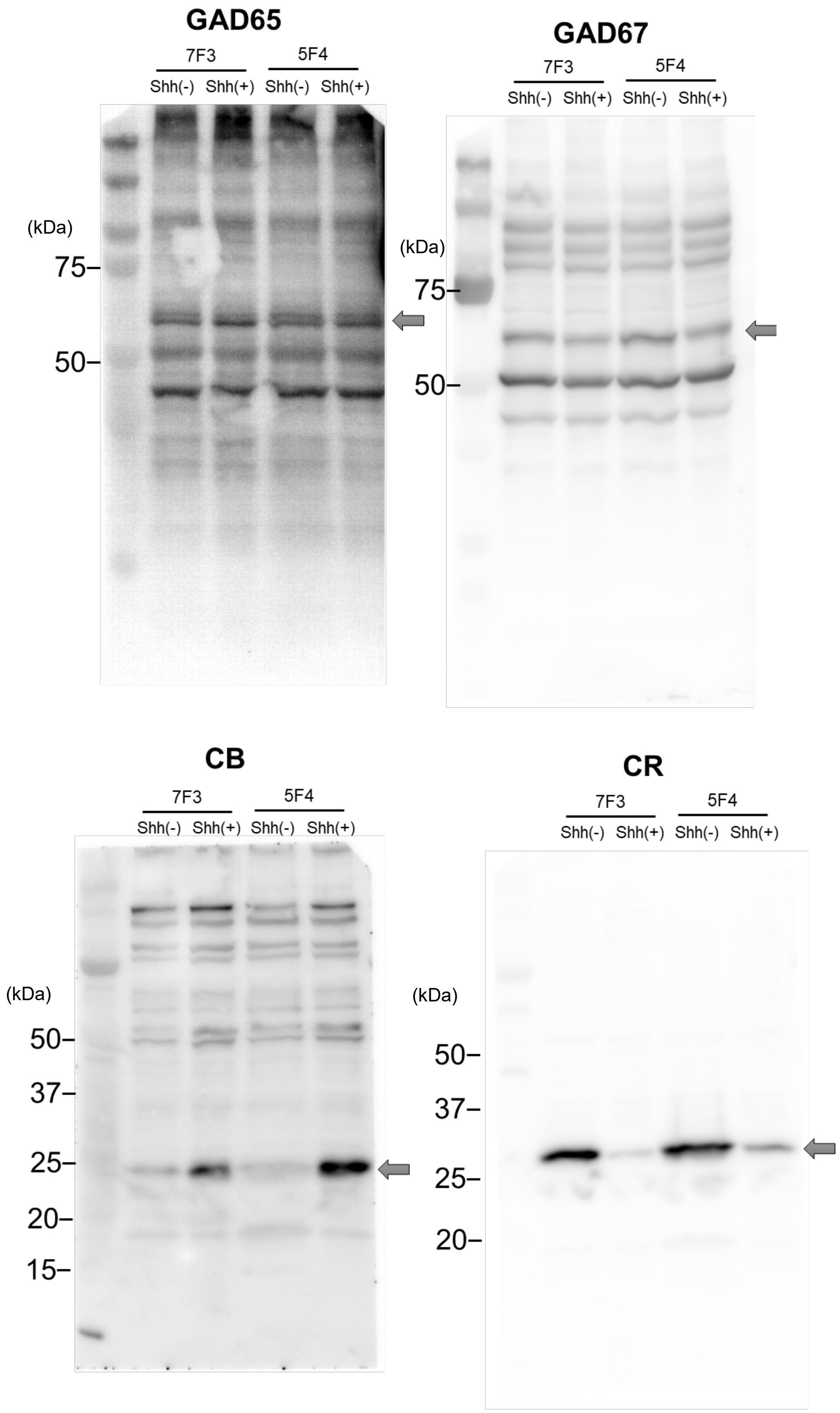

Supplementary Figure 13. Full immnoblot images for Fig. 10.
